# *Entamoeba histolytica* Dmc1 Catalyzes Homologous DNA Pairing and Strand Exchange That Is Stimulated by Calcium and Hop2-Mnd1

**DOI:** 10.1371/journal.pone.0139399

**Published:** 2015-09-30

**Authors:** Andrew A. Kelso, Amanda F. Say, Deepti Sharma, LeAnna L. Ledford, Audrey Turchick, Christopher A. Saski, Ada V. King, Christopher C. Attaway, Lesly A. Temesvari, Michael G. Sehorn

**Affiliations:** 1 Department of Genetics and Biochemistry, Clemson University, Clemson, South Carolina, United States of America; 2 Clemson University Genomics and Computational Biology Laboratory, Institute for Translational Genomics, Clemson, South Carolina, United States of America; 3 Department of Biological Sciences, Clemson University, Clemson, South Carolina, United States of America; 4 Clemson University School of Health Research, Clemson, South Carolina, United States of America; 5 Eukaryotic Pathogens Innovation Center, Clemson University, Clemson, South Carolina, United States of America; 6 Center for Optical Materials Science and Engineering Technologies, Clemson University, Clemson, South Carolina, United States of America; University at Buffalo, UNITED STATES

## Abstract

Meiosis depends on homologous recombination (HR) in most sexually reproducing organisms. Efficient meiotic HR requires the activity of the meiosis-specific recombinase, Dmc1. Previous work shows Dmc1 is expressed in *Entamoeba histolytica*, a eukaryotic parasite responsible for amoebiasis throughout the world, suggesting this organism undergoes meiosis. Here, we demonstrate Dmc1 protein is expressed in *E*. *histolytica*. We show that purified *eh*Dmc1 forms presynaptic filaments and catalyzes ATP-dependent homologous DNA pairing and DNA strand exchange over at least several thousand base pairs. The DNA pairing and strand exchange activities are enhanced by the presence of calcium and the meiosis-specific recombination accessory factor, Hop2-Mnd1. In combination, calcium and Hop2-Mnd1 dramatically increase the rate of DNA strand exchange activity of *eh*Dmc1. The biochemical system described herein provides a basis on which to better understand the role of *eh*Dmc1 and other HR proteins in *E*. *histolytica*.

## Introduction

In most eukaryotic organisms meiosis is essential for reproduction and comprises one round of DNA replication followed by two rounds of cell division to produce haploid gametes. To initiate meiosis, the Spo11 enzyme generates DNA double-strand breaks (DSBs) throughout the genome [1]. The newly formed DSBs are repaired through a DNA repair pathway known as homologous recombination (HR) [2], which forms physical connections between homologous chromosomes. The linkage between homologous chromosomes helps to ensure proper segregation of paired chromosomes at meiotic prophase I. Once the DSB forms, both ends of the DSB are processed to produce 3' ssDNA tails that, in turn, become the nucleation site for the Rad51 and Dmc1 recombinases—two *Escherichia coli* RecA orthologs [3–7]. After binding the ssDNA tail, Rad51 and Dmc1 form right-handed helical nucleoprotein filaments known as presynaptic filaments [8–11]. The presynaptic filament searches for homology by invading the homologous chromosome. When homology is located, Rad51 and Dmc1 catalyze homologous DNA pairing and displace the complementary strand. The resulting structure is known as a displacement loop, or D-loop. Formation of the D-loop is followed by DNA strand exchange. Most organisms express Rad51 ubiquitously, while Dmc1 is expressed only during meiosis. Normal meiosis relies on both Rad51 and Dmc1 [12]. During meiosis, *Saccharomyces cerevisiae* Rad51 serves as an accessory cofactor promoting Dmc1-mediated recombination [13]. Deletion of *DMC1* in *S*. *cerevisiae* results in meiotic arrest in prophase I [14], while *DMC1-/-* knockout mice remain viable but sterile [15]. These results indicate a conserved role for Dmc1 in meiotic recombination [14, 15].

The activity of Dmc1 is modulated by accessory factors such as Rad54B [9], Mei5-Sae3 [16, 17], Swi5-Sfr1 [18], Rad51AP1 [19], and Hop2-Mnd1 [20]. Hop2-Mnd1 is a meiosis-specific heterodimeric protein complex that interacts with Dmc1 to promote the formation of D-loops. Hop2-Mnd1 stabilizes the Dmc1 presynaptic filament that recruits the dsDNA to be searched for homology [21]. Murine Hop2-Mnd1 (mHop2-Mnd1) has been shown to interact and function with human RAD51 [22] and human DMC1 [21]. Additionally, owing to sequence conservation among recombinases, mHop2-Mnd1 was reported to interact with and promote *Schizosaccharomyces pombe* Dmc1- and Rad51-mediated D-loop formation [23].

*Entamoeba histolytica* is a protozoan parasite that causes amoebiasis, which can manifest as amebic dysentery and liver abscesses in more than 50 million humans a year—with approximately 70,000 annual deaths worldwide [24–26]. *E*. *histolytica* has a two-stage life cycle. In the first stage, amoeboid trophozoites proliferate in the colon and may cause disease. As a result of unknown cues, the trophozoite enters the second stage of the life cycle, encystation, which is characterized by genome duplications and formation of tetra-nucleated, environmentally-stable cysts. The cyst-stage facilitates spread to new hosts in contaminated food and water. After ingestion by the host, tetra-nucleated metacystic amoebae emerge in the small intestine (excystation) and undergo several divisions to yield eight trophic amoebae [27–29]. Very little is known about the encystation and excystation processes in *E*. *histolytica* as no axenic encystation condition exists [30]. Therefore, *Entamoeba invadens*, a reptilian parasite that encysts *in vitro*, has been used as a model.

In many organisms, meiosis often results in transmission of gametes or zygotes in order to locate new environments or in the case of parasites, a new host [31]. Several pathogens (*Leishmania* [32], *Trypanosoma brucei* [33–35], and *Giardia lamblia* [36]) undergo meiosis, while *E*. *histolytica* is thought to be asexual. Several lines of evidence suggest that meiosis may occur in *E*. *histolytica*. First, *E*. *histolytica* possesses most of the RAD52 epistasis group of DNA repair genes (RAD50, RAD51, RAD52, RAD54/RDH54, RAD55, RAD57, RAD59, MRE11 AND XRS2) involved with HR, which are highly conserved among eukaryotes [37, 38]. These genes are differentially expressed in response to DNA damage [37]. Second, Singh *et al*. [39] employed an *in vivo* PCR-based method, using inverted repeats, to demonstrate that HR occurs in *E*. *histolytica* and *E*. *invadens*. Third, trophozoites of *E*. *histolytica* and *E*. *invadens* [40, 41] have one nucleus, while cysts have four nuclei, which suggests meiosis occurs during encystation [42]. Fourth, genes known to be involved with meiotic HR, including Dmc1, were identified in *E*. *histolytica* [42]. Lastly, these meiotic genes were expressed [39, 42] during developmental transitions and the formation of the tetra-nucleated cysts, providing support for the idea that meiosis occurs in *E*. *histolytica*.

In the current study, we report for the first time the purification of the *eh*Dmc1 recombinase and the biochemical characterization of its recombination activities. We demonstrate that *eh*Dmc1 forms presynaptic filaments. These filaments are competent to search for homology in duplex DNA to promote ATP-dependent homologous DNA pairing and DNA strand exchange over at least several thousand base pairs. We show that calcium and mHop2-Mnd1 separately enhance *eh*Dmc1 D-loop formation and DNA strand exchange. Importantly, the rate of these recombination activities was significantly increased when both calcium and mHop2-Mnd1 were present. We present evidence that the small molecule, 4,4′-diisothiocyanostilbene-2,2′-disulfonic acid (DIDS), inhibits D-loop formation by *eh*Dmc1. Taken together, our data reveal that *eh*Dmc1 is a functional recombinase providing support for the idea that meiosis occurs in *E*. *histolytica*.

## Materials and Methods

### DNA Substrates

**ϕ**X174 viral (+) strand (ssDNA) and **ϕ**X174 replicative form I (dsDNA) were purchased from New England Biolabs. The **ϕ**X174 replicative form I was digested by *Apa*LI (New England Biolabs) to generate linearized dsDNA. The supercoiled pBluescript DNA was purified from *E*. *coli*, according to manufacturer instructions using a commercially available Giga kit (Qiagen). All oligonucleotides were purchased from Integrated DNA Technologies. The oligonucleotides used in each assay were gel purified using denaturing polyacrylamide gel electrophoresis as described [43]. For the DNA binding assay, the H3 oligonucleotide (ssDNA) was 5'-radiolabeled with [^32^P-**γ**]-ATP using T4 polynucleotide kinase [43]. To obtain the double-stranded substrate (dsDNA), ^32^P-H3 was annealed to the unlabeled complementary H3c oligonucleotide. In the strand exchange assay, OL83-1 was used as the single-stranded substrate, and the double-stranded duplex DNA was generated by radiolabeling OL83-1, as described above, and annealing it with the unlabeled complementary oligonucleotide, OL83-2. The oligonucleotide OL90 was radiolabeled as described above and was used in the D-loop assay and nuclease protection assay. All previously mentioned oligonucleotides sequences can be found in Table 1.

**Table 1:** List of oligonucleotides.

Name	Purpose	Sequence (5'→3')
Primer 1	*eh*Dmc1 Forward	CATCATCATGGAGGAACTGAGGTGAAAAGTAAAAC
Primer 2	His Tag Forward	CATATGCATCATCATCATCATCATGGAGG
Primer 3	*eh*Dmc1 Reverse	GGATCCTTAATCTTTAGCATCAATAATTCCACC
Primer 4	*eh*Rad51 Forward	CATCATCATGGAGGAAGTGCCAAGCAAATAC
Primer 5	*eh*Rad51 Reverse	GGATCCTTAATCATCTTTAACATCTTCAATCCC
H3	DNA binding	TTGATAAGAGGTCATTTGAATTCATGGCTTAGAGCTTAATTGCTGAATCTGGTGCTGGGATCCAACATGTTTTAAATATG
H3c	DNA binding	CATATTTAAAACATGTTGGATCCCAGCACCAGATTCAGCAATTAAGCTCTAAGCCATGAATTCAAATGACCTCTTATCAA
OL83-1	Strand exchange	AAATGAACATAAAGTAAATAAGTATAAGGATAATACAAAATAAGTAAATGAATAAACATAGAAAATAAAGTAAAGGATATAAA
OL83-2	Strand exchange	TTTATATCCTTTACTTTATTTTCTATGTTTATTCATTTACTTATTTTGTATTATCCTTATACTTATTTACTTTATGTTCATTT
OL90	D-loop/ NPA	AAATCAATCTAAAGTATATATGAGTAAACTTGGTCTGACAGTTACCAATGCTTAATCAGTGAGGCACCTATCTCAGCGATCTGTCTATTT

Primers 1–5 were used to isolate and modify the cDNA encoding *E*. *histolytica DMC1* and *RAD51*. H3, OL83-1, and OL90 were ^32^P-radiolabeled using [^32^P-**γ**]-ATP and T4-PNK. ^32^P-H3 and ^32^P-OL83-1 were annealed with H3c and OL83-2 oligonucleotides, respectively, to form double-stranded DNA substrates. ^32^P-OL90 was used in the D-loop and nuclease protection assay.

### Strain and Culture Conditions

*E*. *histolytica* trophozoites (strain HM-1:1MSS) were cultured axenically in TYI-S-33 medium [44] in 15-ml glass screw-cap tubes at 37°C.

### Isolation and Modification of the Genes Encoding *E*. *histolytica DMC1* and *RAD51*

Neither the *E*. *histolytica DMC1* gene (XM_651488) nor the *RAD51* (XM_648984) gene have apparent introns. Thus, it was possible to isolate *DMC1* and *RAD51* cDNA directly from genomic DNA by nested-PCR. Total genomic DNA was isolated from trophozoites using the Wizard Genomic DNA Purification Kit (Promega). The first round of PCR used genomic DNA as a template, and Primers 1 and 3 (Table 1) for *DMC1* and Primers 4 and 5 (Table 1) for *RAD51* isolation. These primers allowed for the incorporation of nucleotides encoding three histidines, a flexible linker of two glycine residues between the tag and the *DMC1* or the *RAD51* coding sequence, and a 3' *BamH*I site. A second round of PCR using the first-round PCR products as templates, and Primers 2 and 3 (Table 1) for *DMC1* and Primers 2 and 5 (Table 1) for *RAD51*, facilitated the integration of nucleotides encoding three additional N-terminal histidines (to form the final N-terminal 6 histidine tag) and a 5' *Nde*I restriction site. The resulting PCR products for both *DMC1* and *RAD51* were digested with *Nde*I and *BamH*I and ligated separately into pET-11a (Novagen). The fidelity of PCR and correctness of the DNA constructs were confirmed by DNA sequencing.

### Expression and Purification of *eh*Dmc1 and *eh*Rad51

The pET-*eh*Dmc1-(HIS)_6_ expression plasmid was introduced into the BL21 *DE3* Rosetta (Novagen) strain of *E*. *coli*. The cells were grown at 37°C until an OD_600_ of ~1.0, and protein expression was induced by the addition of 0.4 mM IPTG at 16°C for 20 hr. The cells were harvested by centrifugation. The cell paste (40 g) was resuspended in Buffer A (50 mM Tris-HCl pH 7.5, 1 mM EDTA, 10% sucrose, 0.01% Igepal, 1 mM β-mercaptoethanol, 0.1 mg/ml lysozyme, 1 mM benzamidine, 1 mM PMSF, and protease inhibitors at a final concentration of 5 μg/ml: aprotinin, chymostatin, leupeptin, and pepstatin A) containing 250 mM KCl. All subsequent steps were performed at 4°C. The resuspended cells were lysed by sonication, and the extract was clarified by ultracentrifugation at 40,000 rpm (Beckman Ti-45 rotor) for 90 min. The clarified lysate was diluted 1:3 in Buffer B (20 mM KH_2_PO_4_ pH 7.5, 10% glycerol, 1 mM EDTA, 1 mM DTT) and loaded onto a 40 ml Q Sepharose column (GE Healthcare). The column was washed with Buffer B containing 100 mM KCl followed by a linear gradient of Buffer B containing 0–800 mM KCl. The peak fractions (~250 mM KCl) containing *eh*Dmc1 were pooled and incubated with 2 ml of Ni-NTA Sepharose (GE Healthcare). The bound protein was eluted with 6 ml of Buffer B containing 500 mM imidazole and 300 mM KCl. The eluate was diluted 1:4 in Buffer B and loaded onto a 1 ml Source 15S column (GE Healthcare). The column was washed with Buffer B containing 100 mM KCl. A linear gradient of Buffer B containing 0–800 mM KCl was applied to the column. The peak fractions containing *eh*Dmc1 (~300 mM KCl) were pooled and loaded onto a 1 ml Source 15Q column (GE Healthcare). Following a wash, the column was subjected to a linear gradient of Buffer B containing 0–700 mM KCl. Peak fractions (~350 mM KCl) containing *eh*Dmc1 were pooled, concentrated, and stored in small aliquots at -80°C. The yield of purified *eh*Dmc1 was ~1 mg. Three independent preparations yielded the same results in the biochemical experiments. *eh*Rad51 was expressed from pET-*eh*Rad51-(HIS)_6_ and purified using the same protocol described for *eh*Dmc1, except that the Q Sepharose column was omitted.

### Expression and Purification of mHop2-Mnd1

The mHop2-Mnd1 expression plasmid was a kind gift from Dr. Daniel Camerini-Otero (National Institute of Health, Bethesda, MD). The mHop2-Mnd1 protein complex was purified as described [20].

### Expression and Purification of hRPA

The expression and purification of the human single strand DNA binding protein, replication protein A (RPA) was performed as described [9, 45].

### Western Analysis

*E*. *histolytica* cells (50 ml, ~4 x10^7^ cells) were pelleted and re-suspended in 10 ml of Buffer A, followed by two freeze-thaw cycles. Glass beads (1 ml; 0.5 mm, BioSpec Products) were added to the resuspended cells followed by vortexing for 3 min. The lysate was centrifuged, and the supernatant was transferred to a new tube. The clarified supernatant was then mixed with 0.25 ml of SP Sepharose (GE Healthcare) followed by gentle rocking at 4°C for 2 hr. The beads were then washed with 0.5 ml of Buffer B containing 100 mM KCl. The bound proteins were eluted with 1 ml of Buffer B containing 500 mM KCl. The eluate was TCA-DOC precipitated, and the precipitation product (15 μl), along with purified *eh*Dmc1 (1 μg) and *eh*Rad51 (1 μg), was subjected to 12% SDS-PAGE followed by transfer to nitrocellulose membrane (Whatman). Rabbit antibody against *sc*Rad51 was a kind gift from Dr. Patrick Sung (Yale University, New Haven, CT) and was used at 1:2000. For the secondary antibody, commercially available HRP-conjugated anti-rabbit IgG (Sigma Aldrich) was used (1:5000), and the membrane was developed using SuperSignal West Pico Chemiluminescent Substrate (Thermo Scientific).

### ATP Hydrolysis Assay

Purified *eh*Dmc1 (1 μM) was incubated with increasing amounts of [^32^P-**γ**]-ATP in 10 μl reaction mixture with Buffer C (20 mM Tris-HCl pH 7.5, 2.4 mM MgCl_2_, 50 mM KCl, 1 mM DTT) at 37°C. After 60 min of incubation, 1.5 μl aliquots were removed, and the reactions were stopped by the addition of an equal volume of 0.5 M EDTA. The products were then subjected to thin-layer chromatography (TLC) using polyethyleneimine-cellulose (PEI) plates (Sigma Aldrich). The amount of ATP hydrolysis was determined using a phosphorimager (Typhoon FLA 7000, GE Healthcare). The ATP hydrolysis assay to determine saturating concentrations of DNA was processed as described above, except *eh*Dmc1 was incubated with the saturating concentration of [^32^P-**γ**]-ATP at (1.5 μM) in the absence or presence of increasing concentrations of **ϕ**X174 (+) virion ssDNA (30 μM, 90 μM, and 120 μM nucleotides) or linearized **ϕ**X174 replicative form I dsDNA (15 μM, 45 μM, and 60 μM base pairs). The time course analysis was performed with a saturating concentration of [^32^P-**γ**]-ATP (1.5 μM) in the presence of ssDNA (90 μM nucleotides) or dsDNA (45 μM base pairs). The DIDS (4,4′-diisothiocyanostilbene-2,2′-disulfonic acid) ATPase assay was processed the same as above, except the addition of DIDS (66.6 μM) with *eh*Dmc1 (0.5 μM) in the presence and absence of **ϕ**X174 ssDNA (90 μM nucleotides). Reactions were processed after 60 min and analyzed as described above.

### Oligonucleotide Electrophoretic Mobility Shift Assay

Increasing amounts of *eh*Dmc1 (1.3 μM, 2.6 μM, 3.9 μM, and 5.2 μM) were incubated with ^32^P-radiolabeled H3 substrate (0.05 pmol) at 37°C in 10 μl of the reaction Buffer D (25 mM Tris-HCl, 2.5 mM ATP, 3 mM MgCl_2_) containing 0.1 mg/ml BSA and 1 mM DTT for 20 min. Increasing amounts of *eh*Dmc1 (5.2 μM, 10.4 μM, 20.8 μM, 31.2 μM) were incubated with dsDNA composed of ^32^P-H3 annealed to H3c using the same experimental conditions as above. A control reaction for both substrates was deproteinized via treatment with SDS (0.5%) and Proteinase K (0.5 μg/ml). The reaction products were subjected to 12% native polyacrylamide gel electrophoresis. The gels were dried on Whatman cellulose chromatography paper (Sigma-Aldrich), and the results were analyzed using a phosphorimager. The DIDS electrophoretic mobility shift assay (EMSA) was processed the same as above, except for the addition of increasing amounts of DIDS (2.5 μM, 5 μM, 7.5 μM, 10 μM, 33.3 μM, 66.7 μM, and 100 μM) with *eh*Dmc1 (5.2 μM) and ^32^P-radiolabeled H3 substrate (0.05 pmol) at 37°C for 20 min. The reaction products were analyzed as described above.

### D-loop Assay

^32^P-radiolabeled OL90 oligonucleotide (3.5 μM nucleotides) was incubated in the presence or absence of *eh*Dmc1 (1.5 μM) in Buffer E (25 mM Tris-HCl, 2 mM ATP, 2.4 mM MgCl_2_) containing 0.1 mg/ml BSA, 1 mM DTT, and the ATP regeneration system consisting of 16 mM creatine phosphate and 36 μg/ml creatine kinase in a final reaction volume 12.5 μl for 10 min at 37°C. The reaction was initiated by the addition of supercoiled pBluescript (35 μM base pairs). At indicated time points, the reactions were deproteinized with the addition of SDS (0.5%) and Proteinase K (0.5 μg/ml). The reaction products were separated using 0.9% agarose gel electrophoresis, dried on DE81 anion exchange paper (GE Healthcare), and analyzed with a phosphorimager. Where indicated, ADP and the ATP analogues, ATP-**γ**-S and AMP-PNP, were used with *eh*Dmc1 in lieu of ATP in the reaction. In the order of addition D-loop assay, the order in which ssDNA and dsDNA were added to the reaction was altered for two reactions. Specifically, (a) *eh*Dmc1 was incubated at 37°C with dsDNA (pBluescript) for 10 min prior to the addition of ssDNA (^32^P-OL90) and (b) *eh*Dmc1 was incubated at 37°C with both ssDNA (^32^P-OL90) and dsDNA (pBluescript) for 10 min. The time course D-loop reactions with calcium and mHop2-Mnd1 were processed as described above except ssDNA (^32^P-OL90) was incubated at 37°C in the presence or absence of *eh*Dmc1 (1.5 μM) for 5 min prior to the addition of CaCl_2_ (0.32 mM) and/or mHop2-Mnd1 (0.16 μM). After an additional 5 min incubation at 37°C, the reaction was initiated by the addition of dsDNA (pBluescript). The reactions were stopped at the indicated time points, as described above. The DIDS (4,4′-diisothiocyanostilbene-2,2′-disulfonic acid) D-loop assay was processed the same as above, except for the addition of increasing amounts of DIDS (2.5 μM, 5 μM, 7.5 μM, and 10 μM) after 2 min of *eh*Dmc1 incubation at 37°C with ssDNA (^32^P-OL90). After 8 min of incubating, the reaction was initiated with the addition of pBluescript followed by a 12 min incubation. The reactions were deproteinized and the products were analyzed as described above.

### Nuclease Protection Assay

^32^P-radiolabeled OL90 (3 μM nucleotides) was incubated with *eh*Dmc1 (1.5 μM) for 1, 2, 5, and 10 min at 37°C in 10 μl total reaction volume in Buffer D. DNase I (2 units; Promega) was added to the reaction followed by an additional 15 min incubation at 37°C. The reaction was deproteinized by treatment with SDS (0.5%) and Proteinase K (0.5 μg/ml) at 37°C for 15 min. The products were subjected to 12% native polyacrylamide electrophoresis, and the gels were analyzed with a phosphorimager. When different ATP analogues and ADP were used, the reactions were performed under the same experimental conditions as above, except ATP-γ-S, ADP, and AMP-PNP (all at 2.5 mM) were substituted for ATP. The DIDS nuclease protection assay was processed the same as above, except for the incubation of increasing concentrations of DIDS (10 μM, 33.3 μM, 66.7 μM, and 100 μM) with *eh*Dmc1 (1.5 μM) and ^32^P-OL90. The reactions were deproteinized, and the products were analyzed as described above.

### Pull-down Assay

The mHop2-Mnd1 complex (3.5 mg) and BSA (5 mg) were immobilized on 0.5 ml of Affi-gel 15 matrix (BioRad) as per the manufacturer instructions. The mHop2-Mnd1-Affi-gel beads or BSA-Affi-gel beads (17.5 μl) were incubated with *eh*Dmc1 (7 μg) in Buffer B with 50 mM KCl. The reactions were incubated at 4°C with mixing for 45 min. After incubating, the supernatant was removed, and the beads were washed three times with Buffer B. An equal volume of SDS loading dye (160 mM Tris-HCl pH 6.8, 60% glycerol, 4% SDS (w/v)) was added to the beads to elute the bound protein. Aliquots of the supernatant, wash, and bead fractions were separated using 12% SDS-PAGE. The gel was visualized by staining with Coomassie blue.

### Oligonucleotide DNA Strand Exchange Assay

The unlabeled OL83-1 oligonucleotide (10 μM nucleotides) was incubated with *eh*Dmc1 (6 μM) for 10 min at 37°C in a 10 μl reaction of Buffer D containing 0.1 mg/ml BSA, 1 mM DTT, and the previously described ATP regeneration system, in the absence or presence of CaCl_2_ (2 mM) and/or mHop2-Mnd1 (0.6 μM). This incubation was followed by the addition of duplex DNA (^32^P-OL83-1/OL83-2; 5 μM base pairs) and spermidine (4 mM final). At the indicated time points, the reactions were stopped by treatment with SDS (0.5% final) and Proteinase K (0.5 μg/ml) at 37°C for 15 min. The products were subjected to 12% native polyacrylamide gel electrophoresis. The gels were analyzed using a phosphorimager.

### Plasmid Length DNA Strand Exchange Assay

Purified *eh*Dmc1 (12.5 μM) was mixed with ϕX174 virion ssDNA (30 μM nucleotides) in Buffer E containing the ATP regeneration system, described above, for 10 min at 37°C followed by the addition of RPA (3.8 μM) and KCl (150 mM final). After an additional incubation for 8 min at 37°C, the linearized double-stranded ϕX174 DNA (30 μM base pairs) and spermidine (4 mM final) were added to the reaction. The reactions were stopped at the indicated time points with the addition of SDS (0.5% final) and Proteinase K (0.5 μg/ml) followed by a 20 min incubation at 37°C. The deproteinized samples were separated on 0.9% agarose gel and stained with ethidium bromide. The gels were analyzed using Image Lab software (BioRad).

### Sequence Alignment and Phylogenetic Classification

Reference Dmc1 amino acid sequences were downloaded from the reference protein database at GenBank (http://www.ncbi.nlm.nih.gov/) and aligned using MUSCLE [46] with 20 maximum iterations. A neighbor-joining tree was prepared using the Jukes-Cantor [47] genetic distance model and edited in Geneious v6.1.6 (www.geneious.com).

### Statistical Analysis

For each assay described, at least three experiments were performed and the error bars represent standard error of the mean.

## Results

### Cloning, Expression, and Purification of *eh*Dmc1

**Fig 1:**
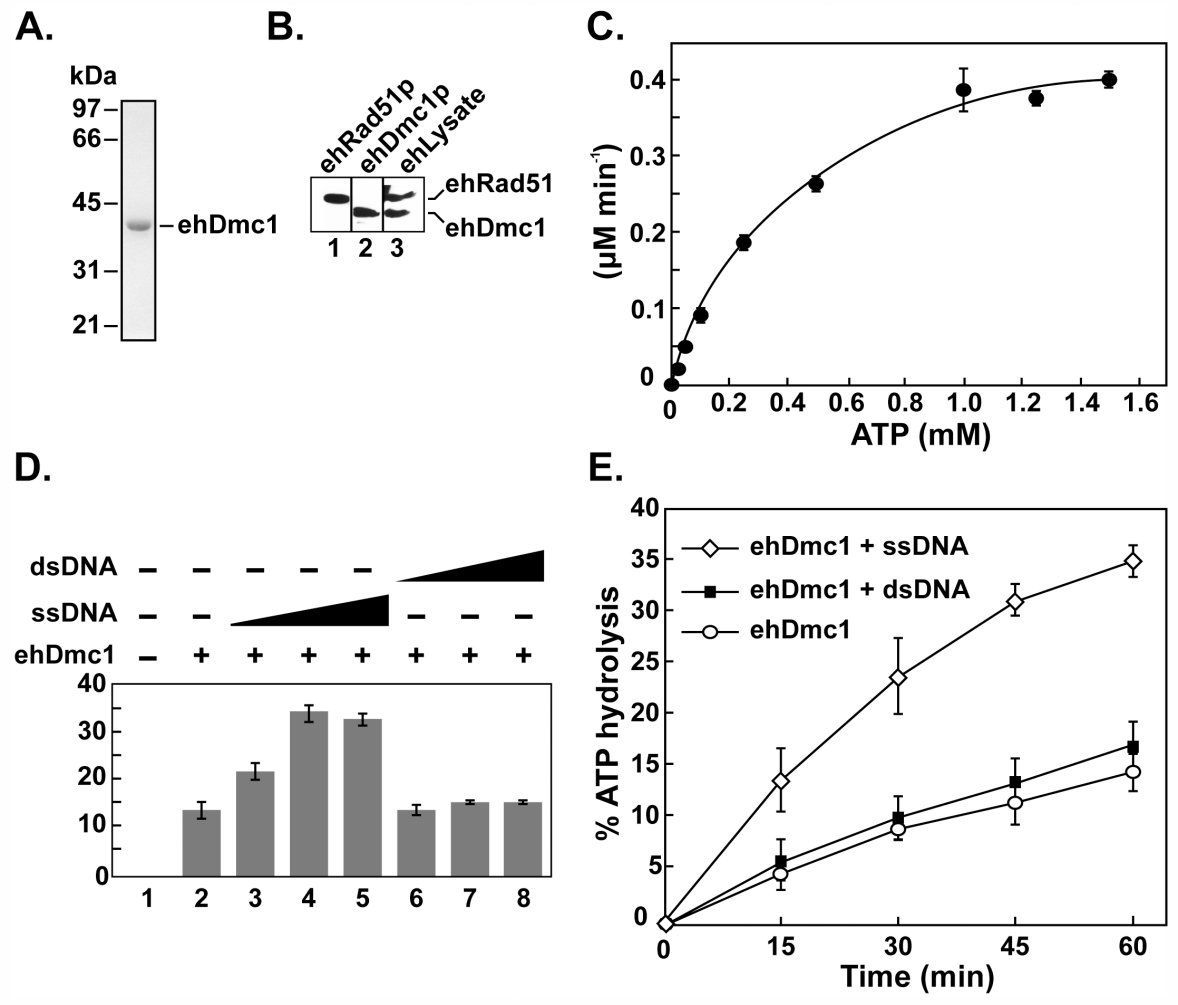
The *eh*Dmc1 and *eh*Rad51 proteins are present in *E*. *histolytica*, and purified *eh*Dmc1 hydrolyzes ATP. **A.** Purified *eh*Dmc1 (~1 μg) on a 12% SDS-polyacrylamide gel stained with Coomassie blue. **B.** Immunoblot of purified recombinant *eh*Rad51 protein and *eh*Dmc1 protein (~1 μg, lane 1 and 2, respectively), and *E*. *histolytica* partially purified lysate (lane 3) on a 12% SDS-polyacrylamide gel. Anti-*sc*Rad51 primary antibodies were used. **C.** Purified *eh*Dmc1 was incubated with increasing concentrations of [^32^P-**γ**]-ATP. After 60 min, samples were withdrawn and the reaction products were separated using TLC followed by analysis with a phosphorimager. **D**. Increasing concentrations of **ϕ**X174 (+) virion single-stranded DNA (ssDNA) or linearized **ϕ**X174 double-stranded DNA (dsDNA) were incubated with *eh*Dmc1 and a saturating concentration of [^32^P-**γ**]-ATP. **E.** Time course analysis of *eh*Dmc1 ATP hydrolysis activity in the absence or presence of **ϕ**X174 ssDNA or linearized **ϕ**X174 dsDNA. Error bars represent SEM, (n = 3).

The presence of the *DMC1* meiotic recombinase gene in the genome of *E*. *histolytica* suggested that *E*. *histolytica* may undergo meiotic recombination. To determine if the putative *ehDMC1* gene encoded a functional recombinase, the *DMC1* gene encoding a 334 amino acid protein was amplified from *E*. *histolytica* genomic DNA and a six-histidine epitope tag was fused to the 5' end of the gene. The gene was sequenced to confirm the absence of any PCR-generated mutations and inserted into the pET11 bacterial expression plasmid. The resultant pET11-*eh*Dmc1-(HIS)_6_ expression plasmid was introduced into bacteria and the cells were induced to produce *eh*Dmc1-(HIS)_6_ protein. We purified *eh*Dmc1 to greater than 95% homogeneity (Fig 1A) using a combination of nickel affinity chromatography and ion exchange chromatography.

In most eukaryotic organisms, Dmc1 is expressed exclusively during meiosis. One exception is the upregulation of Dmc1 in response to radiation-induced mitotic catastrophe (MC) in human cell lines [48] creating a pseudomeiotic state. Considering *E*. *histolytica* is polyploid, we asked whether *eh*Dmc1 protein was expressed in *E*. *histolytica*. Unfortunately, antibodies specific to *eh*Dmc1 are not available commercially. This led us to use a strategy previously reported by Kant *et al*. (2005) that used heterologous antibodies raised against *Saccharomyces cerevisiae* Dmc1 to confirm the identity of the Dmc1 protein from the rice plant, *Oryza sativa* [49]. Previous experience with *sc*Rad51 antibodies revealed a cross-reaction with both hRAD51 and hDMC1, likely due to high sequence conservation (data not shown). Based on the cross-reactivity of the *sc*Rad51 antibodies with hRAD51 and hDMC1, we reasoned the high sequence conservation (61%) between *eh*Dmc1 and hDMC1 may be sufficient for the *sc*Rad51 antibodies to detect *eh*Dmc1. To test this idea, we first asked whether the *sc*Rad51 antibodies recognized highly purified recombinant *eh*Rad51 and *eh*Dmc1. As shown in Fig 1B, *sc*Rad51 antibodies recognized purified recombinant *eh*Rad51 (~40.3 kDa, lane 1) and *eh*Dmc1 (~37.1 kDa, lane 2) confirming that the *sc*Rad51 antibodies cross-reacted with the *eh*Rad51 and *eh*Dmc1 protein. Using the *sc*Rad51 antibodies, we performed Western analysis on partially purified lysate from *E*. *histolytica*. Our results show two bands were detected in lysate from *E*. *histolytica* (Fig 1B, lane 3) that correspond to the same molecular weights of the purified *eh*Rad51 and *eh*Dmc1 (Fig 1B). This suggested that *E*. *histolytica* expresses both recombinase proteins at the same time. These results are in agreement with previous reports that *eh*Rad51 and *eh*Dmc1 mRNA transcripts are present during normal cell culture [37, 42].

### *eh*Dmc1 Hydrolyzes ATP

Analysis of the primary sequence of *eh*Dmc1 revealed conserved Walker A and B motifs (S1 Fig) [50] suggesting the potential for ATP hydrolysis activity. In support of this, both hDMC1 and *sc*Dmc1 proteins harbor a Walker A motif and possess ATP hydrolysis activity [9, 51–53]. To determine if *eh*Dmc1 possessed ATP hydrolysis activity, we performed classic Michaelis-Menten analysis of the ATP hydrolysis activity of *eh*Dmc1. The *eh*Dmc1 protein was incubated with increasing concentrations of [^32^P-**γ**]-ATP followed by TLC. From three independent experiments, a relatively weak ATP hydrolysis activity (*k*_cat_ = 0.53 min^-1^, K_m_ = 480 +/- 50 μM) was detected (Fig 1C). Previous reports indicated the ATP hydrolysis activity of both hDMC1 and *sc*Dmc1 is stimulated by the presence of DNA [9, 52, 53]. We first determined saturating concentrations of ssDNA and dsDNA to be used in the ATP hydrolysis assay (Fig 1D, 90 μM nucleotides and 45 μM base pairs, respectively). We asked if *eh*Dmc1 ATP hydrolysis activity was stimulated in the presence of DNA by incubating the *eh*Dmc1 protein with saturating concentrations of [^32^P-**γ**]-ATP in the absence or presence of saturating amounts of ssDNA or dsDNA prior to TLC. Similar to both hDMC1 and *sc*Dmc1, *eh*Dmc1 ATPase activity was stimulated by both dsDNA and ssDNA, with the greatest stimulation occurring in the presence of ssDNA (Fig 1E). The turnover rate (*k*_cat_) for *eh*Dmc1 ATP hydrolysis was 0.53 min^-1^ which is similar to that reported for hDMC1 (*k*_cat_ = 0.6 min^-1^, [9, 43] and *sc*Dmc1 (*k*_cat_ = 0.7 min^-1^, [52]). It is interesting to note that the turnover rate for *eh*Dmc1, hDMC1 and *sc*Dmc1 are all over 7-fold greater than hRAD51 (*k*_cat_ = 0.07 min^-1^, [54]) and 35-fold greater than *ec*RecA (0.015 min^-1^, [55–57]). The catalytic efficiency (*k*_cat_/K_m_) for *eh*Dmc1 (18 s^-1^ M^-1^) is 1.6 fold greater than hRAD51 (11 s^-1^ M^-1^, [54]).

### DNA Binding Activities of *eh*Dmc1

**Fig 2:**
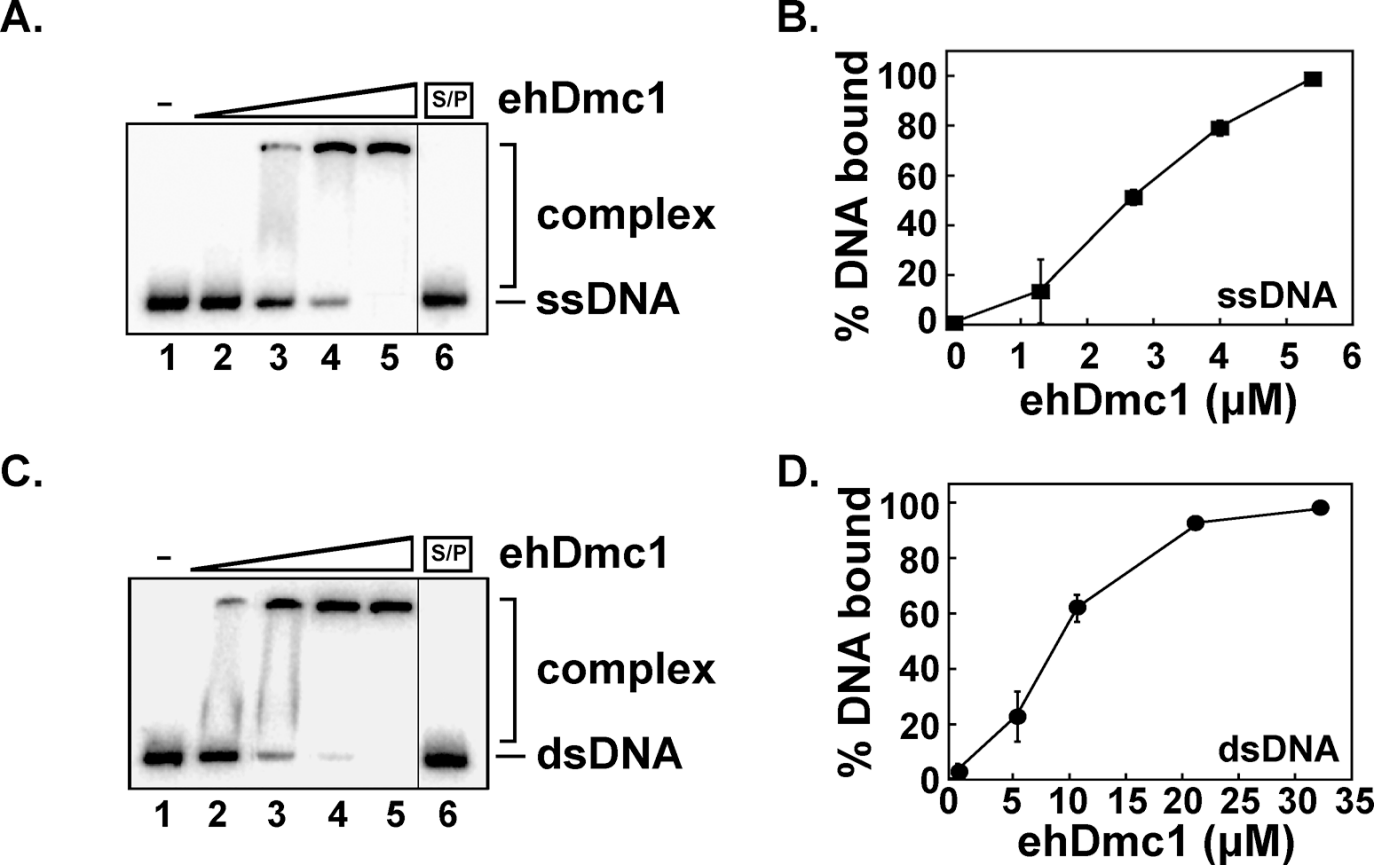
*eh*Dmc1 binds DNA. **A.** Increasing concentrations of *eh*Dmc1 (1.3 μM, lane 2; 2.6 μM, lane 3; 3.9 μM, lane 4; and 5.2 μM, lane 5) were incubated with ssDNA (^32^P-labeled H3 ssDNA). **B.** The mean binding percentages were graphed for three independent experiments from **A**. Error bars represent SEM. **C.** Increasing concentrations of *eh*Dmc1 (5.2 μM, lane 2; 10.4 μM, lane 3; 20.8 μM, lane 4; and 31.2 μM, lane 5) were incubated with dsDNA (^32^P-labeled H3 annealed to H3c). **D.** The mean binding percentages were graphed for three independent experiments from **C**. Error bars represent SEM. Lane 1 for **A** and **C** is devoid of protein, and lane 6 for **A** and **C** was SDS/PK (S/P) treated containing the highest concentration of *eh*Dmc1.

The observation that the ATPase activity of *eh*Dmc1 was stimulated by the presence of DNA suggested that *eh*Dmc1 binds DNA. To demonstrate that *eh*Dmc1 bound DNA, an EMSA was performed. A ^32^P-radiolabeled 80-mer oligonucleotide was used as a ssDNA substrate or was annealed to an unlabeled complementary 80-mer oligonucleotide to create a dsDNA substrate. Increasing concentrations of the *eh*Dmc1 protein were incubated with ssDNA (Fig 2A and 2B) or dsDNA (Fig 2C and 2D) substrates, and the reactions were resolved on a polyacrylamide gel. Fig 2 shows *eh*Dmc1 bound to ssDNA with approximately 5-fold greater affinity than to dsDNA. These results show that *eh*Dmc1 had a strong preference for ssDNA over dsDNA, similar to hDMC1 [43].

### Presynaptic Filament Formation by *eh*Dmc1

**Fig 3:**
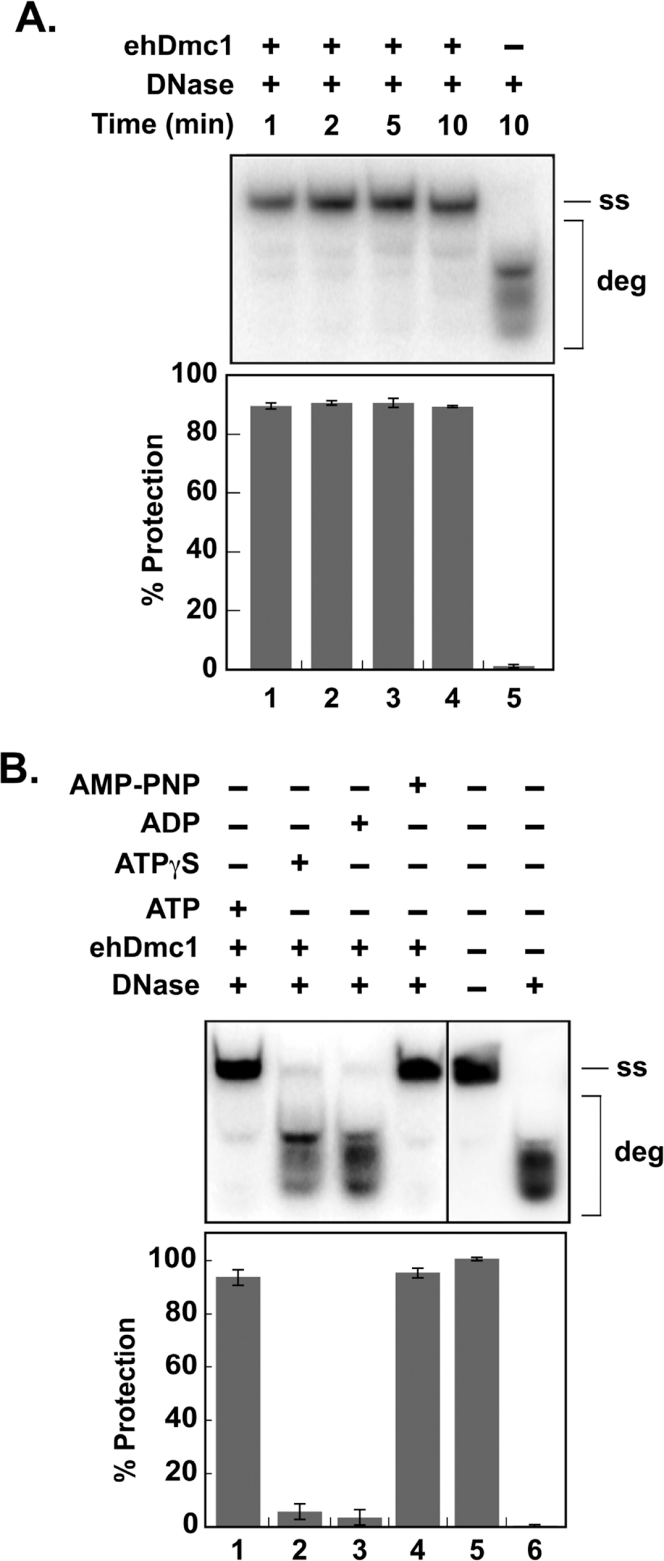
The *eh*Dmc1 nucleoprotein filament protects ssDNA in the presence of DNase I. **A.** ^32^P-radiolabeled OL90 ssDNA was incubated with *eh*Dmc1 prior to the addition of DNase I. At the indicated times, an aliquot was removed and deproteinized. The reaction products were separated on a 12% native polyacrylamide gel followed by analysis with a phosphorimager. The mean percent protection of the ssDNA from DNase I digestion of three independent experiments was graphed. Error bars represent SEM. Lane 5 is devoid of protein. **B.** ^32^P-OL90 ssDNA was incubated with *eh*Dmc1 in the presence of 2.5 mM nucleotide (ATP, lane 1; ATP-γ-S, lane 2; ADP, lane 3; and AMP-PNP, lane 4) prior to the addition of DNase I. Lane 5 is devoid of protein and DNase I. Lane 6 is devoid of protein but contains DNase I. After a 10 min incubation, an aliquot was removed and processed as described in **A**. The mean percent protection of three independent experiments was graphed. Error bars represent SEM. (ss), ^32^P-radiolabeled single-stranded OL90; (deg) degradation.

Formation of a nucleoprotein filament is critical for the recombination activities of Dmc1 [9, 51, 58]. Dmc1 forms stacked octameric protein rings on ssDNA in the absence of ATP [9, 51, 59]. These protein rings are not the active form of Dmc1-ssDNA. In the presence of ATP, Dmc1 forms an active right-handed nucleoprotein filament on ssDNA [9]. The difference between the Dmc1 nucleoprotein filament and stacked octameric rings of Dmc1 on ssDNA can be visualized by using a nuclease protection assay [43, 60, 61]. To determine if *eh*Dmc1 formed presynaptic filaments on ssDNA, we used this nuclease protection assay [43, 60, 61]. In this assay, if *eh*Dmc1 forms a nucleoprotein filament, the ssDNA will be protected from nucleolytic digestion by DNase I by the *eh*Dmc1 nucleoprotein filament. hRAD51, *sc*Rad51, hDMC1 and *sc*Dmc1 require ATP binding in order to form a presynaptic filament [8, 9, 62, 63]. Therefore, ^32^P-radiolabeled ssDNA was incubated with *eh*Dmc1 in the presence of ATP to allow for presynaptic filament formation. After a brief incubation, DNase I was added to the reaction. The time course of the DNase I digestion showed that *eh*Dmc1 formed a presynaptic filament that protected the ssDNA from DNase I digestion (Fig 3A) The filament formed within one minute and lasted throughout the course of the assay, suggesting *eh*Dmc1 is capable of forming a rapid and stable presynaptic filament. Next, we wished to determine the nucleotide dependence of *eh*Dmc1 presynaptic filament formation. Using the same nuclease protection assay as described above, we incubated *eh*Dmc1 with ^32^P-radiolabeled ssDNA in the absence of nucleotide or in the presence of ATP, ADP, the slowly hydrolyzable analog ATP-**γ**-S, or the non-hydrolyzable ATP analog AMP-PNP. DNase I was added to the reaction after a brief incubation. When either ATP or AMP-PNP, (Fig 3B, lanes 1 and 4, respectively) was incubated with *eh*Dmc1, the ssDNA was strongly protected from DNase I digestion. Incubation of *eh*Dmc1 with, ATP-**γ**-S, or ADP (Fig 3B, lanes 2 and 3, respectively) resulted in greatly reduced protection of the ssDNA from DNase I digestion. In the absence of ATP, *eh*Dmc1 was unable to protect the ssDNA from DNase I (Fig 3B, lane 6). Taken together, the results suggest that *eh*Dmc1 requires ATP binding but not hydrolysis to form presynaptic filaments.

### *eh*Dmc1 Catalyzes DNA Homologous Pairing

**Fig 4:**
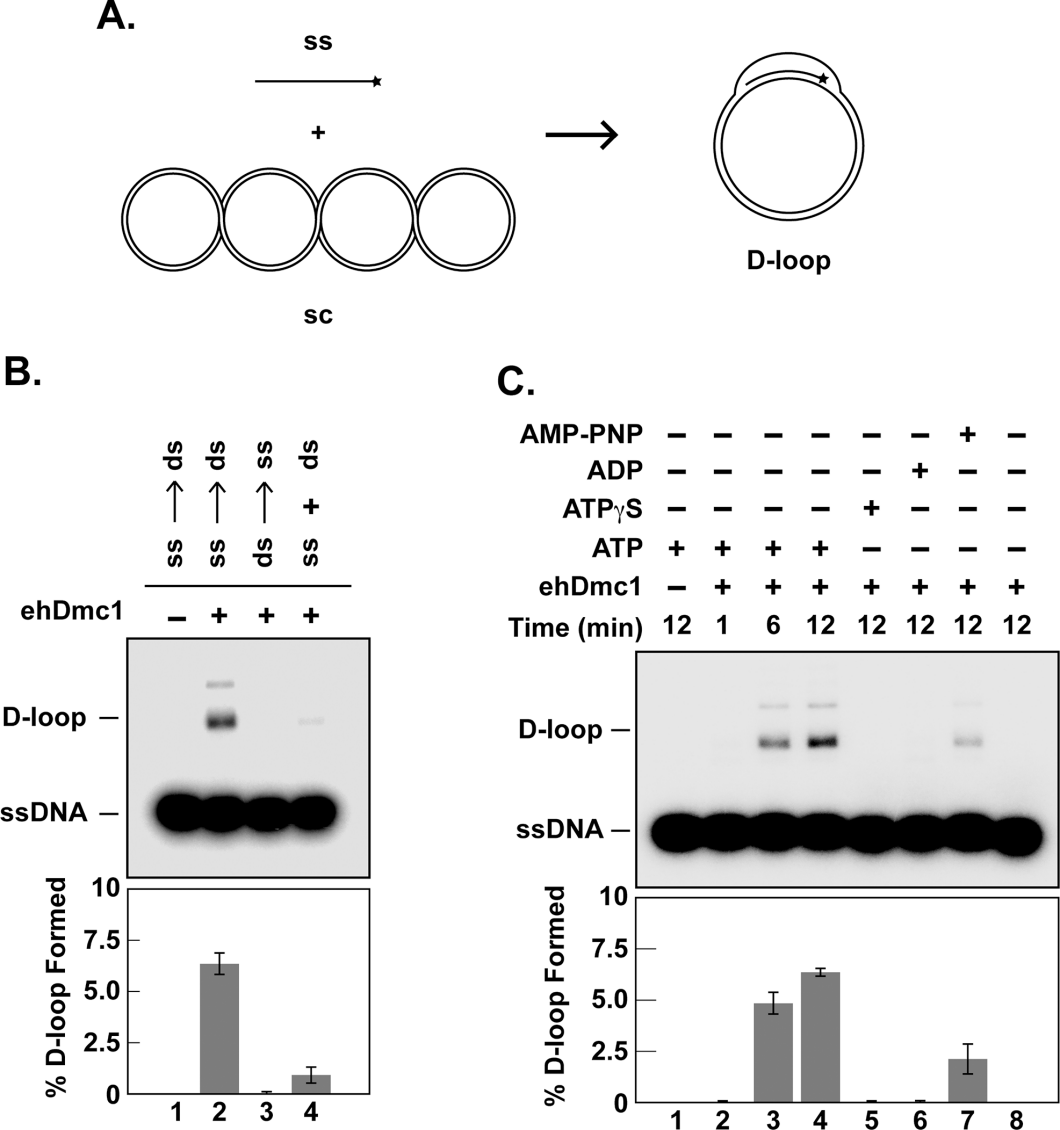
*eh*Dmc1 catalyzes D-loop formation. **A.** Schematic of D-loop formation assay (ss, single-strand oligonucleotide; sc, supercoiled dsDNA). **B.** *eh*Dmc1 was incubated with ^32^P-radiolabeled OL90 ssDNA (lane 2), dsDNA (lane 3) prior to the addition of dsDNA or ssDNA (lanes 2 and 3, respectively), or both ssDNA and dsDNA (lane 4) simultaneously. Lane 1 is devoid of protein. After a 12 min incubation, an aliquot was removed and deproteinized prior to separation on an agarose gel. The mean percent of six independent experiments was graphed. Error bars represent SEM. **C.** *eh*Dmc1 was incubated with ^32^P-OL90 ssDNA in the presence of 2 mM nucleotide (ATP, lanes 1–4), ATP-**γ**-S (lane 5), ADP (lane 6) and AMP-PNP (lane 7). Lane 8 was devoid of nucleotide. At the indicated times, an aliquot was removed and processed as described in **B**. The mean percent of six independent experiments was graphed. Error bars represent SEM.

The ability of *eh*Dmc1 to form a stable presynaptic filament suggested that *eh*Dmc1 may be able to commence homologous DNA pairing. To determine if the *eh*Dmc1 presynaptic filaments mediated DNA homologous pairing, an assay was used that monitors the formation of a D-loop [43]. In this assay, *eh*Dmc1 was incubated with a ^32^P-radiolabeled oligonucleotide (ssDNA) to allow presynaptic filament formation. Upon addition of this nucleoprotein complex to supercoiled duplex DNA harboring a region of complementary sequence, the ^32^P-radiolabeled oligonucleotide was assimilated into a supercoiled duplex DNA through base-pairing with the complementary sequence within the duplex DNA. As a result, the homologous sequence was displaced forming a D-loop (Fig 4A). Guided by previous work on hDMC1 [9], we determined the preferred order of addition for the ssDNA and supercoiled dsDNA. Our results show that *eh*Dmc1 strongly prefers to form a presynaptic filament on ssDNA prior to the addition of duplex DNA (Fig 4B, lane 2). Co-addition of ssDNA and dsDNA resulted in greatly reduced D-loop formation (Fig 4B, lane 4). No D-loop was detected when *eh*Dmc1 was incubated on supercoiled dsDNA prior to the addition of ssDNA (Fig 4B, lane 3). This preference of forming a presynaptic filament on ssDNA to form D-loop is in agreement with that of hDMC1 [9]. To determine if *eh*Dmc1 required ATP hydrolysis for D-loop formation, ATP was substituted with AMP-PNP, ATP-**γ**-S, or ADP. D-loop formation by *eh*Dmc1 was seen in the presence of ATP (Fig 4C, lanes 2–4). In the presence of AMP-PNP, the D-loop formation by *eh*Dmc1 was significantly weaker than that seen in the presence of ATP (Fig 4C, compare lanes 4 and 7). This result is similar to that seen with hDMC1 [43]. Contrastingly, *sc*Dmc1-mediated D-loop formation was reported to be greater in the presence of AMP-PNP than with ATP [52]. Neither ATP-**γ**-S nor ADP supported *eh*Dmc1-mediated D-loop formation (Fig 4C, lanes 5 and 6, respectively). As expected, no D-loop formation was seen in the absence of ATP (Fig 4C, lane 8). These results suggest *eh*Dmc1, like *sc*Dmc1 and hDMC1, requires ATP binding but not hydrolysis to form D-loop [9, 52].

### *eh*Dmc1 Interacts with Murine Hop2-Mnd1

**Fig 5:**
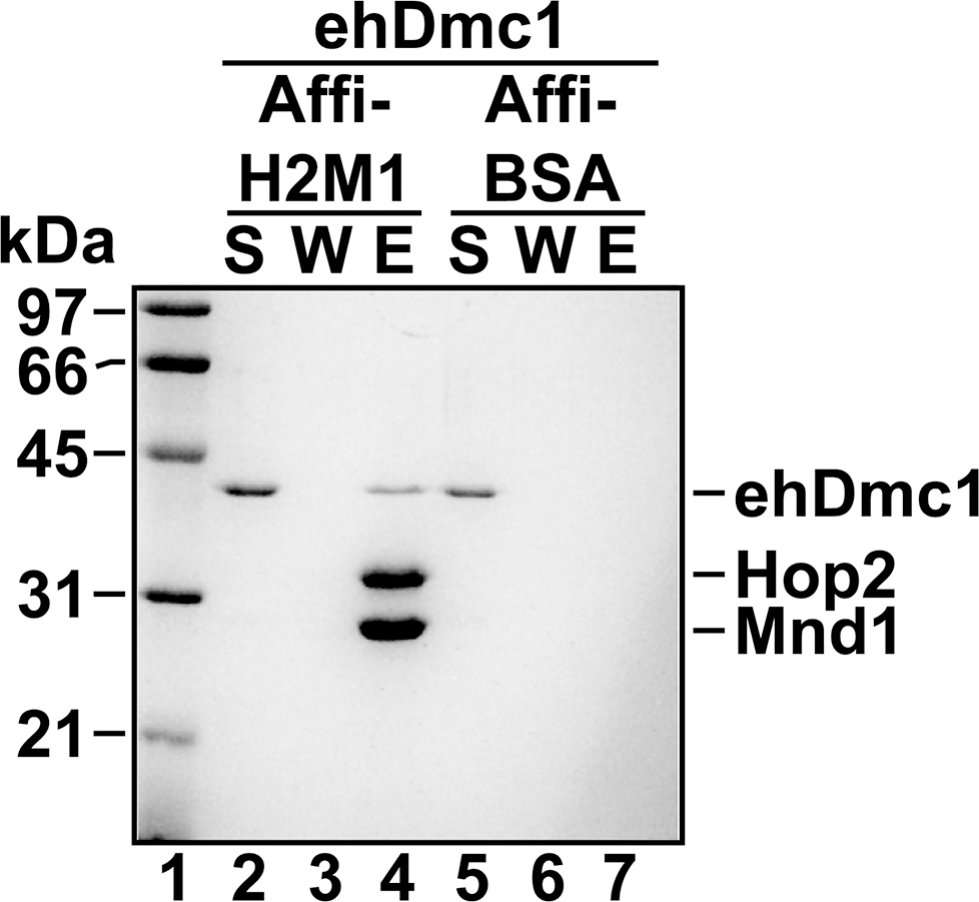
mHop2-Mnd1 interacts with *eh*Dmc1. *eh*Dmc1 was mixed with Affi-Gel matrix conjugated to either mHop2-Mnd1 (lanes 2–4) or bovine serum albumin (BSA, lanes 5–7). After a wash, bound protein was eluted with SDS. The supernatant (S), wash (W), and eluate (E) were subjected to SDS-PAGE, and the gel was stained with Coomassie blue.

The activity of the Dmc1 recombinase is modulated by several accessory factors [2] that include the heterodimeric meiotic recombination accessory protein complex, Hop2-Mnd1. Much of our understanding of the molecular biochemical role of Hop2-Mnd1 in homologous recombination comes from studies that used mHop2-Mnd1 with the hRAD51 and hDMC1 recombinases [20–22, 64–70]. In addition to the interaction with hRAD51 and hDMC1 recombinases, the mHop2-Mnd1 complex was shown to interact with *sp*Dmc1 [23]. This is likely due to the high degree of amino acid conservation between hDMC1 to murine Dmc1 (~97%; mDmc1) and to *sp*Dmc1 (~60% identity). The discovery of a missing exon in the *S*. *cerevisiae* Hop2 gene finally allowed active *sc*Hop2-Mnd1 complex to be purified [71]. While *E*. *histolytica* possess the genes that encode *eh*Hop2 and *eh*Mnd1, purified *eh*Hop2-Mnd1 complex is currently not available. Based on the heterologous interaction between mHop2-Mnd1 and hRAD51, hDMC1 and *sp*Dmc1 [20–23, 67], and given *eh*Dmc1 is ~61% identical to hDMC1, we reasoned that mHop2-Mnd1 may interact with *eh*Dmc1. To determine if *eh*Dmc1 interacted with mHop2-Mnd1, an affinity pull-down assay was performed using Affi-gel matrix conjugated with either mHop2-Mnd1 or bovine serum albumin (BSA). When *eh*Dmc1 was incubated with Affi-mHop2-Mnd1, *eh*Dmc1 was found in the eluate indicating a physical interaction (Fig 5, lane 4). Incubation of *eh*Dmc1 with Affi-BSA beads resulted in *eh*Dmc1 being found only in the supernatant (Fig 5, lane 5) signifying no interaction between *eh*Dmc1 and BSA. The results indicate the interaction between *eh*Dmc1 and mHop2-Mnd1 was specific.

### *eh*Dmc1-mediated D-loop Formation is Stimulated by mHop2-Mnd1 and Ca^2+^

**Fig 6:**
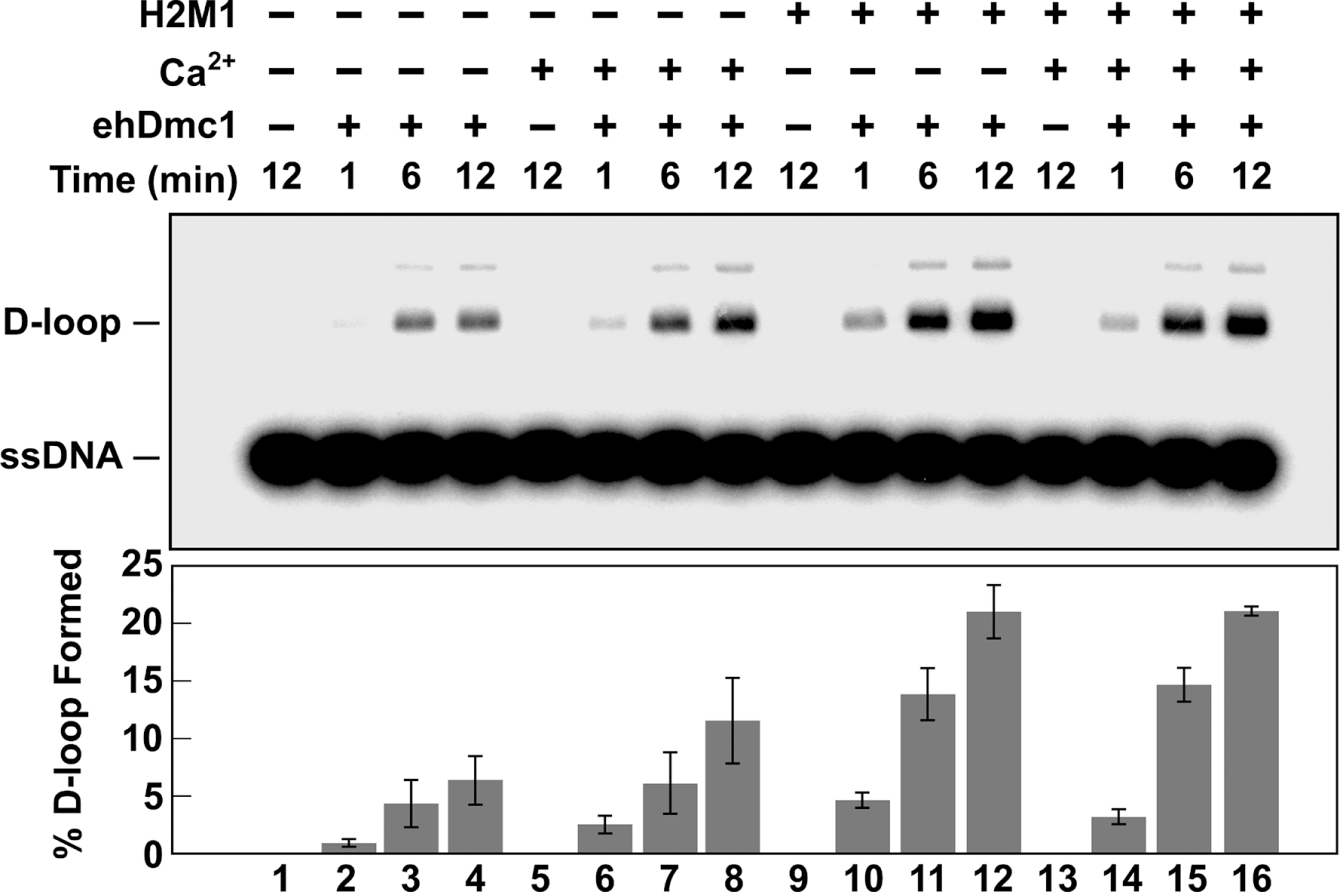
mHop2-Mnd1 and Ca^2+^ stimulate *eh*Dmc1-mediated D-loop formation. *eh*Dmc1 was incubated with ^32^P-radiolabeled OL90 ssDNA in the absence (lanes 1–4 and 9–12) or presence of calcium (lanes 5–8 and 13–16) and/or mHop2-Mnd1 (lanes 9–16). The reaction was initiated with the addition of supercoiled dsDNA. Aliquots were removed at the indicated times, deproteinized, and the reaction products were separated by agarose gel electrophoresis. Lanes 1, 5, 9, and 13 were lacking *eh*Dmc1. Mean values from three individual experiments were graphed. Error bars represent SEM.

Previous reports indicated the D-loop forming activity of *sc*Dmc1 [63, 71, 72] and hDMC1 [51] is enhanced by calcium. Using the preferred order of addition and nucleotide to support *eh*Dmc1-mediated homologous DNA pairing, we used the D-loop assay to determine if calcium stimulated *eh*Dmc1 D-loop formation. Calcium mediated an approximate 2-fold stimulation of D-loop formation catalyzed by *eh*Dmc1 (Fig 6, compare lanes 2–4 with 6–8). Our observation that *eh*Dmc1 interacts with mHop2-Mnd1 and previous reports demonstrating mHop2-Mnd1 enhances both *sp*Dmc1 [23] and hDMC1 D-loop formation [20, 67] led us to ask if mHop2-Mnd1 stimulated *eh*Dmc1 D-loop formation. When mHop2-Mnd1 was added to the *eh*Dmc1 D-loop reaction, there was an approximate 3.5-fold increase in D-loop formation (Fig 6, lanes 10–12). Since both calcium and mHop2-Mnd1 stimulated D-loop formation, we investigated whether co-addition of calcium with mHop2-Mnd1 would further enhance *eh*Dmc1 D-loop formation. As shown in Fig 6 (lanes 14–16), addition of calcium did not further enhance *eh*Dmc1 D-loop formation in the presence of mHop2-Mnd1. Taken together, the results indicate *eh*Dmc1 catalyzes DNA homologous pairing. This activity is stimulated by both calcium and mHop2-Mnd1 in agreement with reports of Dmc1 from other species [20, 23, 67].

### *eh*Dmc1 Catalyzes DNA Strand Exchange Using ϕX174 DNA

**Fig 7:**
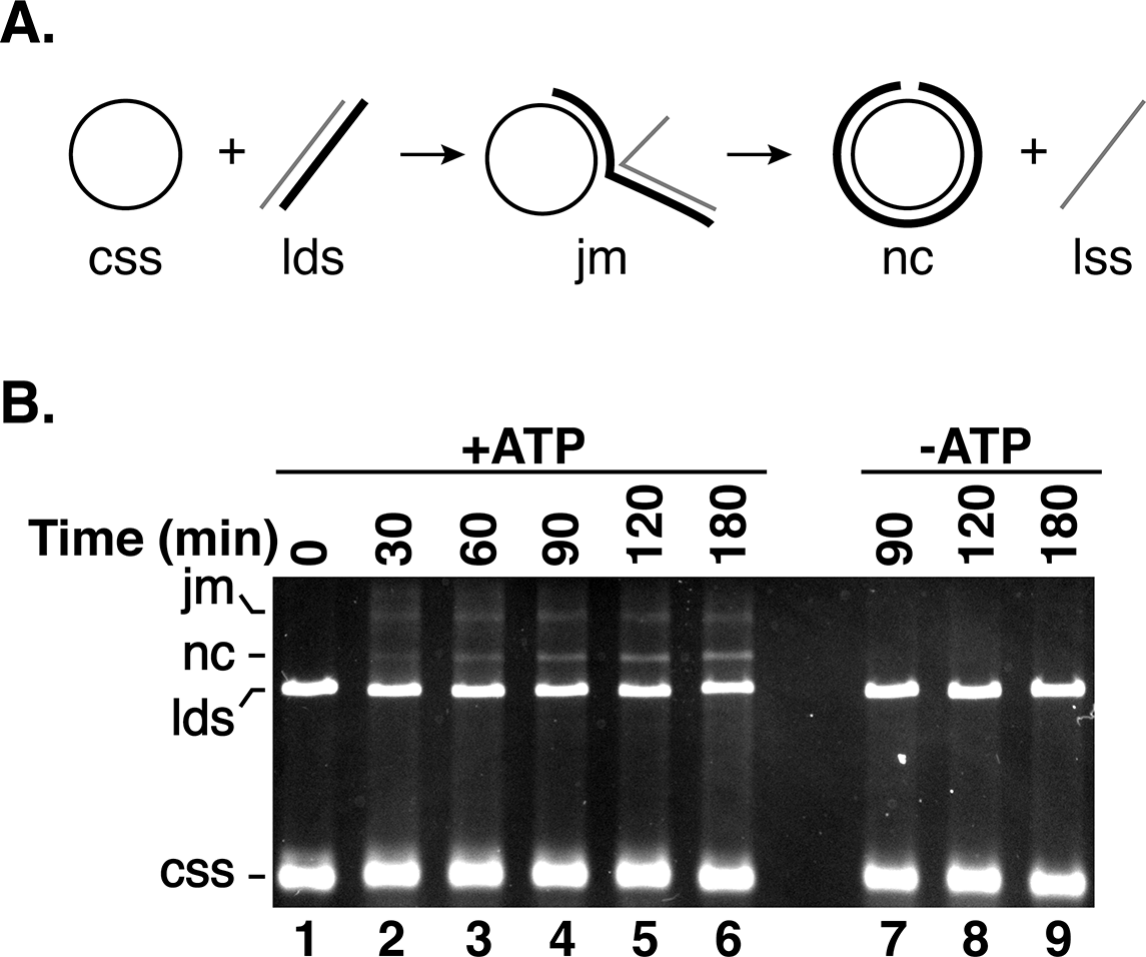
*eh*Dmc1 mediates plasmid length DNA strand exchange. **A.** Schematic of the 3-strand homologous DNA pairing and strand exchange reaction. Homologous DNA pairing between the circular ssDNA (css) and linear dsDNA (lds) first forms a DNA joint molecule (jm). DNA strand exchange converts the joint molecule into a nicked circular duplex (nc) displacing the linear ssDNA (lss). **B.** *eh*Dmc1 (12.5 μM) was incubated with ϕX174 virion ssDNA (css) to allow presynaptic filament formation to occur before the addition of hRPA (3.8 μM) and KCl (150 mM final concentration). The reaction was initiated by the addition of linearized double-strand ϕX174 DNA (lds) and spermidine. At the indicated time points, the reactions were deproteinized, subjected to agarose gel electrophoresis, and stained with ethidium bromide.

The ability of *eh*Dmc1 to form D-loops led us to determine whether *eh*Dmc1 was capable of DNA strand exchange in a 3-strand assay that utilizes **ϕ**X174 DNA viral (+) ssDNA and linearized **ϕ**X174 dsDNA that are 5.4 kilobase pairs in length [9]. In this assay, *eh*Dmc1 was pre-incubated with the **ϕ**X174 DNA viral (+) ssDNA to allow for presynaptic filament formation. This was followed by the addition of hRPA, a single strand DNA binding protein that stabilizes the ssDNA by preventing the formation of secondary structure. The addition of linear **ϕ**X174 dsDNA initiated the reaction leading to joint molecule formation and strand exchange forming a nicked circular product (Fig 7A). As shown in Fig 7B, *eh*Dmc1 catalyzed ATP-dependent DNA strand exchange over 5.4 kilobase pairs. This DNA strand exchange was similar to the activity shown for Dmc1 proteins from other species [9, 18, 73]. Although the molecular effect of spermidine on the ability of other recombinases [9, 74] to commence DNA strand exchange is not known, we found *eh*Dmc1 DNA strand exchange was dependent upon the presence of spermidine (data not shown).

### Oligonucleotide DNA Strand Exchange by *eh*Dmc1

**Fig 8:**
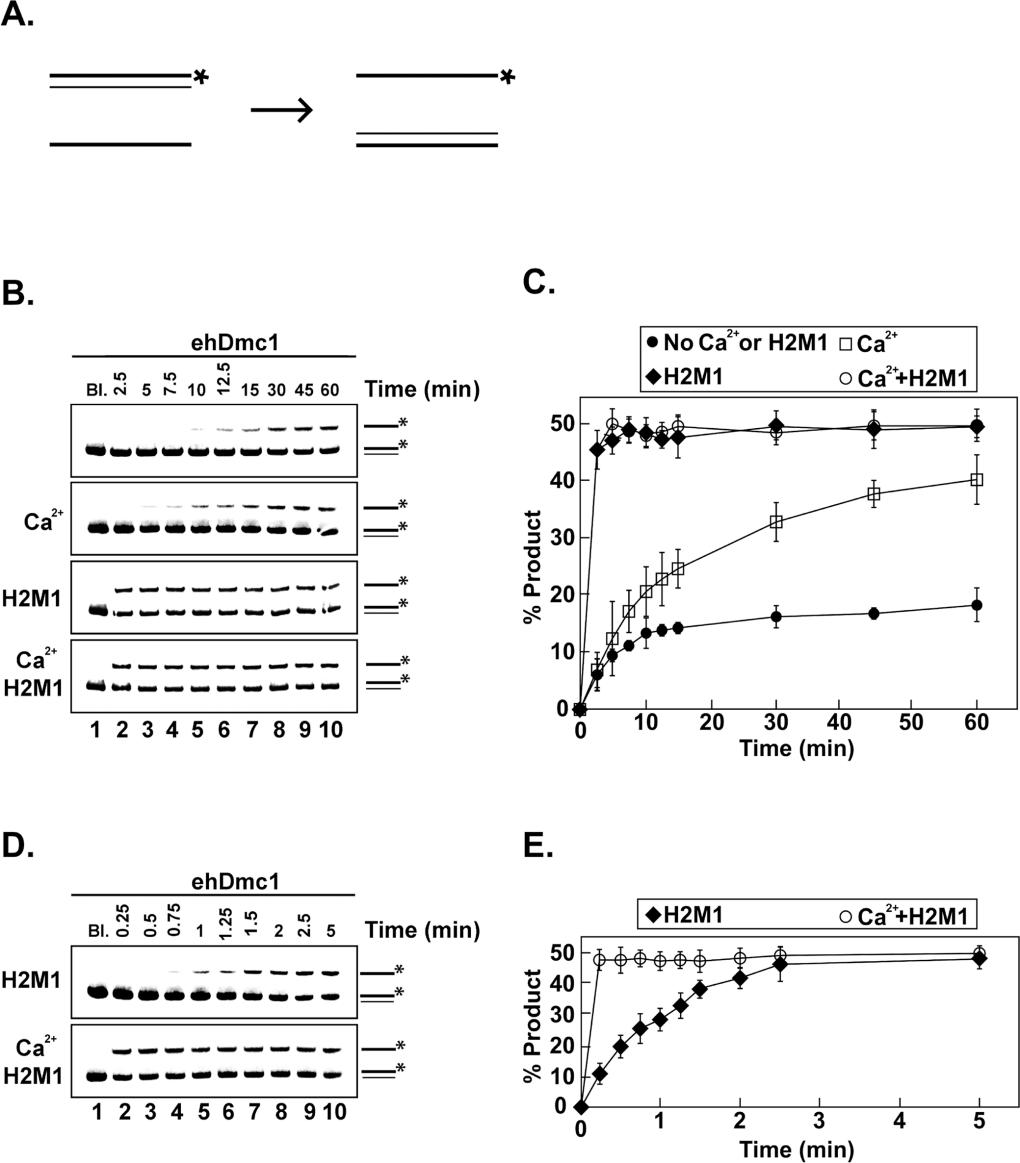
Stimulation of *eh*Dmc1-mediated DNA strand exchange activity by mHop2-Mnd1 and Ca^2+^. **A.** Schematic of oligonucleotide strand exchange assay. **B.** A time course analysis of *eh*Dmc1 strand exchange activity (top panel) in the presence of 2 mM calcium (Ca^2+^), mHop2-Mnd1 (H2M1), and the combination of calcium and mHop2-Mnd1 (Ca^2+^ H2M1), as indicated. At the indicated times, an aliquot was removed and deproteinized. The reaction products were separated on 12% native polyacrylamide gels, and the gels were analyzed by a phosphorimager. Lane 1 is devoid of protein (Bl.). **C.** Mean values from three individual experiments from **B** were graphed. Error bars represent SEM. **D.** A 5 min time course analysis of *eh*Dmc1 strand exchange activity in the presence of mHop2-Mnd1 (H2M1) or the combination of 2 mM calcium and mHop2-Mnd1 (Ca^2+^ H2M1), as indicated. At the indicated times, an aliquot was removed, deproteinized and processed as described in **B**. Lane 1 (Bl.) is devoid of protein. **E.** Mean values of three independent experiments from **D** were plotted. Error bars represent SEM.

Since *eh*Dmc1 weakly catalyzed DNA strand exchange on plasmid length DNA substrates, we switched to an oligonucleotide-based DNA strand exchange assay [43, 45] to further investigate the influence of calcium and mHop2-Mnd1 on *eh*Dmc1. In this assay, an oligonucleotide ssDNA substrate was incubated with *eh*Dmc1 followed by the addition of radiolabeled dsDNA to initiate the reaction. DNA strand exchange occurs when the radiolabeled strand of the dsDNA substrate is replaced by the unlabeled homologous ssDNA within the *eh*Dmc1 presynaptic filament (Fig 8A). Our results demonstrate that *eh*Dmc1 is adept at DNA strand exchange using oligonucleotide substrates (Fig 8). The presence of calcium in the reaction increased not only the rate of DNA strand exchange but also the amount of strand exchange product (~38%) compared to the absence of calcium (~16%). The addition of mHop2-Mnd1 alone or in combination with calcium greatly increased the rate of *eh*Dmc1-mediated DNA strand exchange (Fig 8B and 8C). The time scale of the reactions containing mHop2-Mnd1 separately or in combination with calcium did not allow us to determine if calcium enhanced the *eh*Dmc1/mHop2-Mnd1-mediated DNA strand exchange. Therefore, we performed the same DNA strand exchange assay using shorter time points. The presence of calcium greatly increased the rate of the DNA strand exchange activity of *eh*Dmc1-mHop2-Mnd1 (Fig 8D and 8E) with the reaction reaching a plateau in less than 15 seconds. There was over a 240-fold increase in the rate of the strand exchange reaction mediated by *eh*Dmc1 in the presence of both calcium and mHop2-Mnd1. Taken together, these results indicate that calcium and mHop2-Mnd1 enhance *eh*Dmc1-mediated DNA strand exchange.

### Phylogenetic Relationship of *eh*Dmc1 to Other Pathogens, Yeast, and Higher Eukaryotes

The similarity of the response by *eh*Dmc1 and hDMC1 to calcium and mHop2-Mnd1 in D-loop formation and DNA strand exchange prompted us to evaluate the relationship of *eh*Dmc1 to hDMC1, *sc*Dmc1, *sp*Dmc1, and Dmc1 from other pathogens by sequence alignment (S1 Fig). The phylogenetic tree (S2 Fig) shows that *eh*Dmc1 is more closely related to other pathogens than to the yeasts and human. However, *eh*Dmc1 shares 201 identical sites with the hDMC1 and 176 with *sc*Dmc1 (S1 Fig) with the most unique region of the protein among the groups being the C-terminal domain, suggesting *eh*Dmc1 is more similar to hDMC1 than *sc*Dmc1.

### DIDS Inhibits *eh*Dmc1 Homologous DNA Pairing

**Fig 9:**
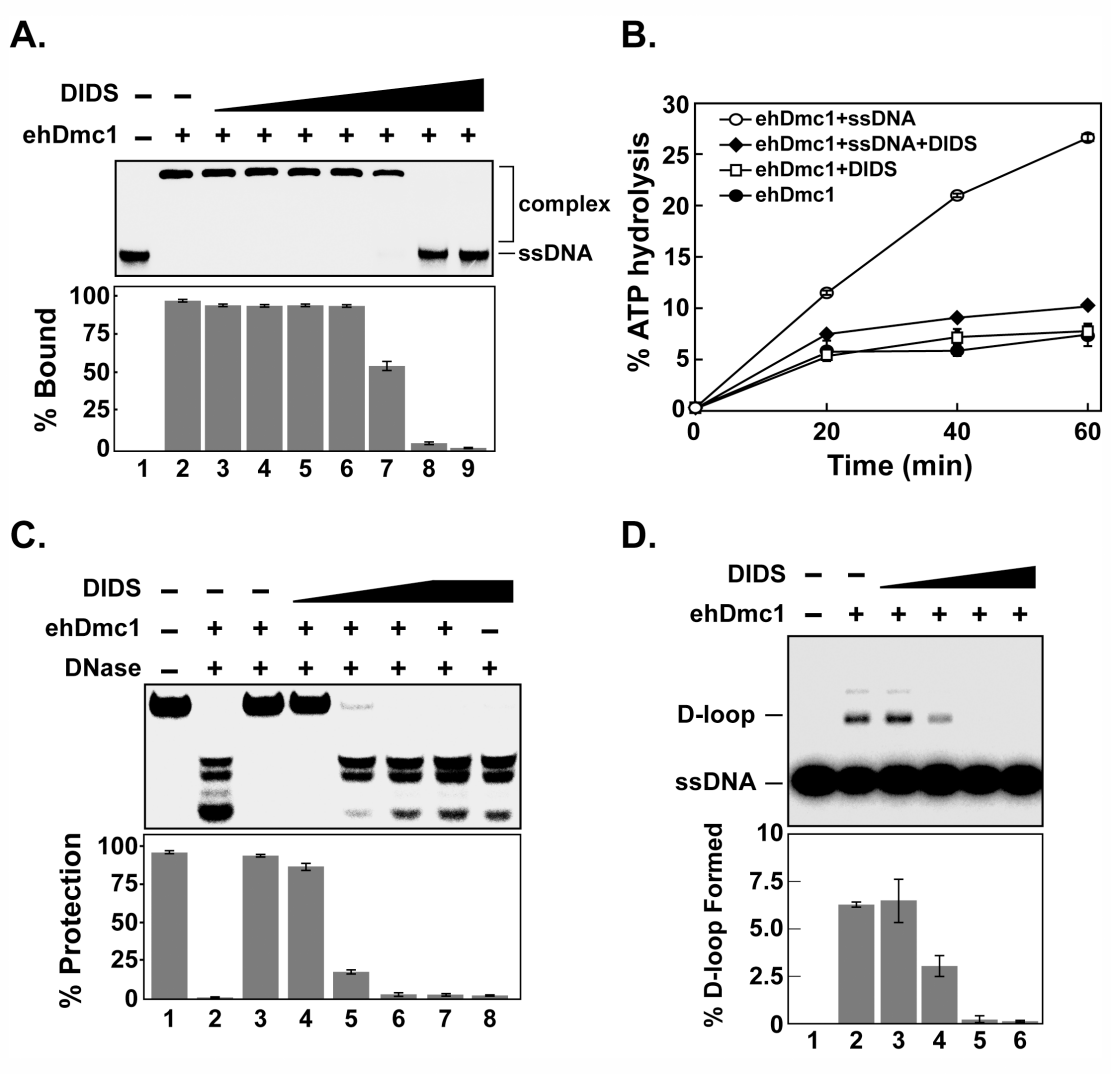
DIDS inhibits presynaptic filament formation by *eh*Dmc1. **A.** *eh*Dmc1 was incubated with ^32^P-radiolabeled OL90 ssDNA in the presence and absence of increasing amounts of DIDS at 37°C for 20 min. Products were separated on 12% polyacrylamide gels and analyzed with a phosphorimager. **B.** *eh*Dmc1 was incubated with saturating amounts of [^32^P-**γ**]-ATP in the presence and absence of **ϕ**X174 ssDNA and/or DIDS (66.6 μM). The reactions were stopped at the indicated times, subjected to TLC, and analyzed using a phosphorimager. **C.** *eh*Dmc1 was incubated with ^32^P-radiolabeled OL90 in the presence and absence of increasing amounts of DIDS followed by exposure to DNase for 15 min at 37°C. The reactions were stopped, separated on 12% polyacrylamide gels, and analyzed with a phosphorimager. **D.** *eh*Dmc1 was incubated with ^32^P-radiolabeled OL90 ssDNA for 2 min prior to the addition of DIDS (2.5 μM, lane 3; 5 μM, lane 4; 7.5 μM, lane 5; and 10 μM, lane 6). After 8 min of incubation, the reaction was initiated by the addition of supercoiled dsDNA. After 12 min, an aliquot was removed and deproteinized. The reaction products were separated by agarose gel electrophoresis, and the gels were analyzed with a phosphorimager. Mean results from three separate experiments were graphed. Error bars represent SEM. DIDS, 4,4'-diisothiocyanostilbene-2,2'-disulfonic acid.

To date, no inhibitors for any Dmc1 protein have been identified. However, several compounds have been reported to inhibit hRAD51 [75]. We tested one of these compounds, 4,4′-diisothiocyanostilbene-2,2′-disulfonic acid (DIDS) to determine if it would inhibit *eh*Dmc1-mediated functions [76, 77]. Previous work suggested DIDS may inhibit DNA binding to the hRAD51 recombinase [76]. We wished to determine if DIDS directly inhibited DNA binding by *eh*Dmc1. To do this, we used an EMSA where *eh*Dmc1 was incubated with ssDNA, ATP and increasing concentrations of DIDS. As shown in Fig 9A, DIDS inhibited ssDNA binding by *eh*Dmc1. The concentration of DIDS that was required to completely inhibit ssDNA binding by *eh*Dmc1 was ~6 fold higher (66 μM) than reported for hRAD51 (10 μM, [76]). Because ATP was present in the EMSA, we wished to determine whether DIDS directly inhibited ssDNA binding by *eh*Dmc1 or whether DIDS indirectly inhibited DNA binding by interfering with ATP binding that is necessary for presynaptic filament formation. We addressed these possibilities using the ATP hydrolysis assay. Incubation of *eh*Dmc1 in the presence of ssDNA with the amount of DIDS that inhibited DNA binding resulted in attenuation of the stimulatory effect ssDNA has on *eh*Dmc1 ATP hydrolysis activity (Fig 9B). In the absence of ssDNA, DIDS had no effect on the ATP hydrolysis activity of *eh*Dmc1 (Fig 9B). These results suggest DIDS directly interferes with ssDNA binding and not with binding of ATP. We used the nuclease protection assay to monitor the stability of the *eh*Dmc1 nucleoprotein filament in the presence of DIDS. *eh*Dmc1 was incubated in the presence or absence of ssDNA and/or increasing concentrations of DIDS. Our results show that DIDS inhibits presynaptic filament formation (Fig 9C). Notably, the concentration of DIDS that inhibits presynaptic filament formation is the same concentration that inhibits ssDNA binding by *eh*Dmc1. Lastly, we asked whether the inhibition of presynaptic filament formation by DIDS would compromise the ability of *eh*Dmc1 to catalyze D-loop formation. The results show that increasing concentrations of DIDS inhibited D-loop formation by *eh*Dmc1 (Fig 9D). The IC_50_ of DIDS on *eh*Dmc1-mediated homologous pairing is 4.5 +/- 0.23 μM. Our results demonstrate that *eh*Dmc1 is a potential target for small molecule inhibitors in a manner similar to hRAD51 [75, 76, 78–83].

## Discussion

Here, we report that *eh*Dmc1 protein is expressed in *E*. *histolytica*. We cloned and devised a purification procedure for the *eh*Dmc1 recombinase. We demonstrated purified *eh*Dmc1 possesses ATP hydrolysis activity that was stimulated preferentially by ssDNA. *eh*Dmc1 binds ssDNA with a higher affinity than with dsDNA and forms presynaptic filaments on ssDNA in an ATP-dependent manner. Additionally, *eh*Dmc1 catalyzed robust ATP-dependent D-loop formation and DNA strand exchange, which is stimulated by the presence of calcium. We showed that mHop2-Mnd1 interacts with *eh*Dmc1 to enhance the D-loop formation and DNA strand exchange activity of *eh*Dmc1. Our results demonstrated that *eh*Dmc1 catalyzes DNA strand exchange over several thousand base pairs. Based on phylogenetic analysis, we found that *eh*Dmc1 is more similar to higher plants and pathogens. Our biochemical and phylogenetic analysis show that *eh*Dmc1 is more similar to hDMC1 than *sc*Dmc1. Lastly, we demonstrated that a small molecule, DIDS, is an effective inhibitor of *eh*Dmc1 homologous DNA pairing. Our data provide strong evidence that *eh*Dmc1 is catalytically active and may be a potential target for therapeutic treatment for *E*. *histolytica* infection.

The co-expression of both Rad51 and Dmc1 recombinases is typically seen only in meiosis [14, 84–86]. We were surprised to find two proteins bands recognized by our *sc*Rad51 antibodies that correspond to the molecular weights of *eh*Dmc1 and *eh*Rad51 in *E*. *histolytica* normal cell culture. Importantly, these two bands migrate similarly in SDS-PAGE with purified recombinant *eh*Dmc1 and *eh*Rad51. We are careful to note we relied on the cross-reactivity of *sc*Rad51 antibodies to detect expression of *eh*Dmc1 and *eh*Rad51. Previously, two bands (41 and 46 kDa) of *eh*Rad51 were observed by Lopez-Casamichana *et al*. [37]. These molecular weights differ from the predicted molecular weight of *eh*Rad51 (*eh*Rad51 is 40.3 kDa containing 366 amino acids and *eh*Dmc1 is 37.1 kDa containing 334 amino acids). The difference in molecular weight of the two protein bands observed in this study and that previously reported [37] were both ~4 kDa. Therefore, like the antibodies used in this study, the antibodies used by Lopez-Casamichana *et al*. [37] may have also cross-reacted with *eh*Dmc1. As a result, Lopez-Casamichana may have actually detected both *eh*Dmc1 and *eh*Rad51.

Our results demonstrate that *eh*Dmc1 has similar biochemical characteristics as those previously described for Dmc1 recombinases from other organisms [9, 18, 23, 51, 52]. The ATPase is stimulated by both ssDNA and dsDNA with ssDNA providing the greater level of stimulation. The preference of binding ssDNA is in agreement with the preferred order of addition in the D-loop formation assay where the recombinase binds to ssDNA prior to the addition of dsDNA. The formation of a stable presynaptic filament is dependent upon the binding and not hydrolysis of ATP as the non-hydrolyzable ATP analog, AMP-PNP, supported presynaptic filament formation to the same extent as ATP. The observation that AMP-PNP did not support the same level of D-loop formation is similar to that reported for hDMC1 [9] but unlike *sc*Dmc1 [72]. The lack of D-loop formation by *eh*Dmc1 in the presence of ATP-**γ**-S is similar to what is reported for hDMC1 [9]. It is not clear why ATP-**γ**-S fails to support D-loop formation or DNA strand exchange by *eh*Dmc1 and hDMC1. However, ATP-**γ**-S weakly supports DNA strand exchange and D-loop formation by hRAD51 [87]. This difference could reflect slight differences within the ATP binding pocket that permit hRAD51 to accommodate ATP-**γ**-S. It is possible, that *eh*Dmc1 has low affinity for ATP-**γ**-S. In support of this idea, hRAD51-ssDNA nucleoprotein filaments formed in the presence of ATP-**γ**-S are less extended than *ec*RecA nucleoprotein filaments [62]. Furthermore, the hRAD51-ssDNA nucleoprotein filaments formed in the presence of ATP-**γ**-S resemble the inactive *ec*RecA nucleoprotein filaments formed in the absence of a nucleotide cofactor [88].

The ATP-dependent DNA strand exchange using plasmid length DNA substrates was rather weak when compared hDMC1 [9], hRAD51 [87], and *sc*Rad51 [74]. We suggest the full potential of *eh*Dmc1 may not be realized due to the use of human single-strand DNA binding protein, replication protein A (RPA) in the 3-strand DNA strand exchange assay.

Our results show that mHop2-Mnd1 physically interacts with *eh*Dmc1. We find this result intriguing given the identity of *eh*Dmc1 to mDmc1 is 61% and to hDMC1 61%. Furthermore, mHop2 has only 32% identity with *eh*Hop2 and mMnd1 has 41% identity with *eh*Mnd1. We interpret these results to suggest that the interaction surfaces between Dmc1 and Hop2-Mnd1 proteins are well conserved. In agreement with this idea, Ploquin *et al*. (2007) demonstrated the *sp*Dmc1 interacted with mHop2-Mnd1. Here, the 32% identity between mHop2 and *sp*Hop2 is the same as that between mHop2 and *eh*Hop2 while the 33% identity between mMnd1 and *sp*Mnd1 is lower than the identity between 41% mMnd1 and *eh*Mnd1. Despite this, both *sp*Dmc1 and *eh*Dmc1 interact with mHop2-Mnd1.

We show that calcium and mHop2-Mnd1 enhance both homologous DNA pairing and DNA strand exchange by *eh*Dmc1. When both calcium and mHop2-Mnd1 were present, we failed to see any further enhancement of *eh*Dmc1-mediated D-loop formation than seen with either calcium or mHop2-Mnd1 alone. This may be due to the selection of time points for the D-loop formation experiments. To resolve this issue, we switched to a slower oligonucleotide-based DNA strand exchange assay [43]. When we initially used the oligonucleotide-based DNA strand exchange assay, the results were similar to those seen in the *eh*Dmc1-mediated D-loop formation assay where no apparent difference between incubation with mHop2-Mnd1 alone or in combination with calcium was observed. However, the use of shorter time points revealed that calcium synergistically worked with mHop2-Mnd1 to dramatically increase the rate of the *eh*Dmc1-mediated DNA strand exchange reaction over 240-fold. Importantly, we show that calcium is not required for mHop2-Mnd1-mediated stimulation of *eh*Dmc1 as reported for *sc*Hop2-Mnd1 and *sc*Dmc1 [71]. These results suggest that the activation of *eh*Dmc1 by mHop2-Mnd1 is mechanistically different than the activation by calcium. In support of this idea, calcium was shown to inhibit the ATP hydrolysis activity of hDMC1 and promote a more stable hDMC1-ADP-ssDNA complex [51], while Hop2-Mnd1 is reported to stabilize the hDMC1 presynaptic filament and facilitate the capture of dsDNA [67]. Our results demonstrating that *eh*Dmc1 interacts with, and is stimulated by mHop2-Mnd1 suggests *eh*Hop2-Mnd1 will likely enhance the recombination activities of *eh*Dmc1. It will be important to demonstrate these putative interactions upon availability of purified *eh*Hop2-Mnd1 protein complex.

*Leishmania* [32], *T*. *brucei* [33–35], and *G*. *lamblia* [36] are pathogens that undergo meiosis. A distantly related amoebazoan, *Dictyostelium discoideum* also has a meiotic cycle [89, 90]. Currently, meiosis is only proposed to occur during encystation in *E*. *histolytica* [42]. Given that HR was demonstrated in *E*. *histolytica* [39], *eh*Dmc1 is expressed in *E*. *histolytica* [42], our results demonstrating the *eh*Dmc1 protein is likely present in *E*. *histolytica* in cell culture, and our biochemical analysis demonstrating *eh*Dmc1 is an active recombinase, provides additional support for the possibility that meiosis occurs in *E*. *histolytica*.

An alternate explanation is that polyploidy in *E*. *histolytica* may be an adaptation for survival. This is seen in human cancer cells that are known to be aneuploid, tetraploid, and polyploid [91, 92]. Radiation treatment of cancer cells induces MC [79] leading to aberrant mitosis. As a result, radiation treated cancer cells become polyploid and aneuploid. Polyploid cells are more resistant to radiation treatment than diploid cells [93]. While polyploidy may be temporarily beneficial to cancer cells, the genome is highly unstable and often triggers DNA checkpoint cell cycle arrest. These cells may attempt to escape cell death through depolyploidization using HR, but most often, the cells undergo apoptosis [94]. However, there are instances of meiotic genes, including Dmc1, being aberrantly upregulated in cancer cells in response to radiation-induced MC [48]. These cells were shown to depolyploidize to become smaller mononucleated cells that survive the radiation treatment and produce progeny [48]. This response to radiation treatment in human cancer cells is similar to the response of *Cryptococcus neoformans* to fluconazole where the cell becomes aneuploid for specific chromosomes [95]. Once the fluconazole is removed, the cells depolyploidize to their original chromosome copy number. It is possible that *E*. *histolytica* does not undergo meiosis, but uses *eh*Dmc1-mediated HR in a manner similar to these radiation-induced MC surviving cancer cells to maintain the pseudomeiotic polyploid state of the *E*. *histolytica* cell in response to unknown cues that induce encystation. These examples suggest the formation of a polyploid or aneuploid state may be a conserved survival tactic utilized by eukaryotic organisms.

We present the first report of a small molecule inhibitor for any Dmc1 recombinase. Our demonstration that DIDS is a small molecule inhibitor of *eh*Dmc1 may provide a helpful tool to reveal if *eh*Dmc1 has a role in encystation or as a potential drug therapeutic. The biochemical system described herein should provide a basis on which to better understand the role of *eh*Dmc1 and other HR proteins in *E*. *histolytica*.

## Supporting Information

S1 Fig Dmc1 multiple sequence alignment. A multiple sequence alignment was constructed with MUSCLE, depicting amino acid sequence similarities and variance. The boxes indicated the two conserved Walker Motifs.
(TIF)

S2 Fig Dmc1 neighbor-joining tree. A phylogenetic tree was constructed from 36 representative taxa that encode a functional Dmc1 with 70 nodes. *eh*Dmc1 shares a higher similarity with other pathogens and higher order plant species. *eh*Dmc1 is more similar to hDMC1 than *sc*Dmc1.
(TIF)
