# Supplementary figures and images for: Entamoeba histolytica Dmc1 Catalyzes Homologous DNA Pairing and Strand Exchange That Is Stimulated by Calcium and Hop2-Mnd1
Source: PLoS One. 2015 Sep 30;10(9):e0139399. doi: 10.1371/journal.pone.0139399 (PMC4589404; doi:10.1371/journal.pone.0139399)

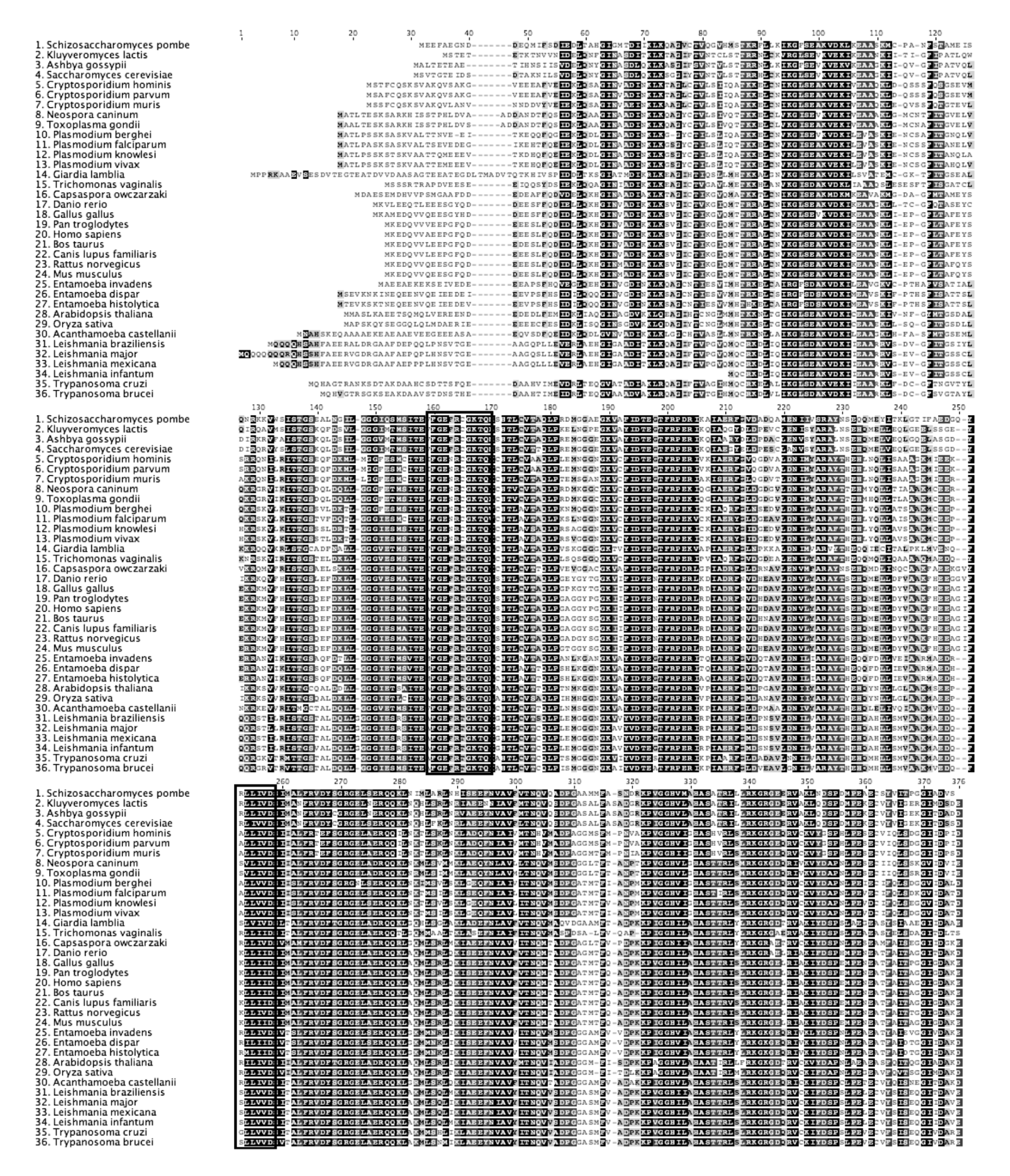

Supplement: S1 Fig — A multiple sequence alignment was constructed with MUSCLE, depicting amino acid sequence similarities and variance. The boxes indicated the two conserved Walker Motifs. (TIF) [file pone.0139399.s001.tif]

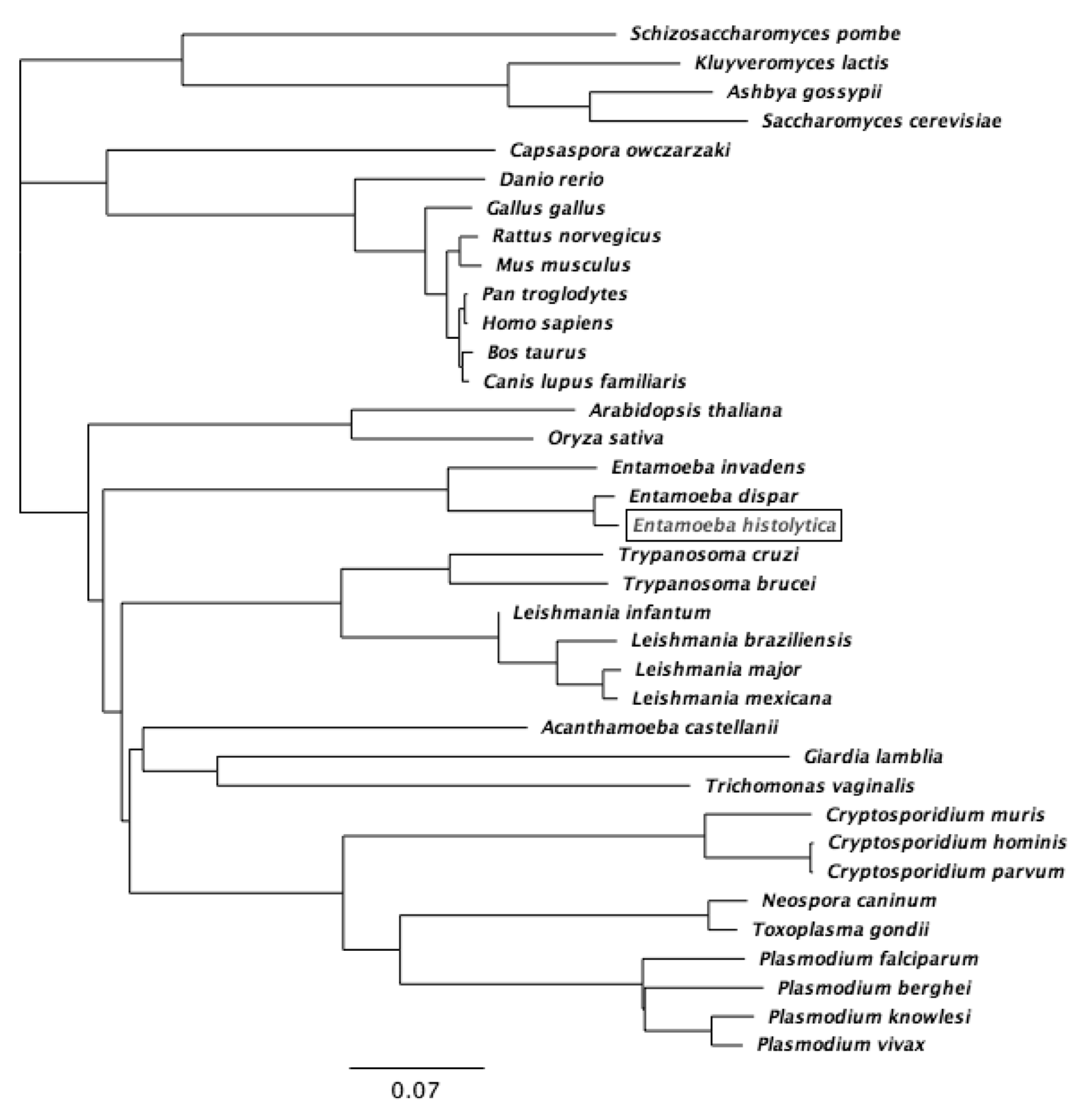

Supplement: S2 Fig — A phylogenetic tree was constructed from 36 representative taxa that encode a functional Dmc1 with 70 nodes. ehDmc1 shares a higher similarity with other pathogens and higher order plant species. ehDmc1 is more similar to hDMC1 than scDmc1. (TIF) [file pone.0139399.s002.tif]
